# Balancing Cognitive Flexibility and Stability: The Role of Reward, Autism, and Transdiagnostic Traits

**DOI:** 10.5334/joc.511

**Published:** 2026-07-24

**Authors:** Leslie K. Held, Judith Goris, Senne Braem

**Affiliations:** 1 Department of Experimental Psychology, Ghent University, Belgium

**Keywords:** task-switching, autism, cognitive flexibility, reward learning, reinforcement

## Abstract

In our daily lives, we often need to switch between states of cognitive flexibility and stability based on environmental demands. It has been suggested that people can learn to navigate this balance based on reinforcement, but that this may be impaired in autism or other transdiagnostic dimensions. Replicating previous work, we show that people (n = 412) voluntarily switch tasks more when previously rewarded more for performing well on task-switch trials. Interestingly, we also found that it was difficult to unlearn this shift in cognitive flexibility when the mapping between task-switching and reward suddenly reversed. People with an autism diagnosis or high autism traits were not slower or faster in learning these contingencies. However, our results suggested a generally smaller task-rule interference effect and larger switch costs in autism and high autism traits, in line with prevalent ideas that they show more cognitive stability at the cost of less flexibility. A similar pattern was found for a significantly correlated Compulsivity and intrusive thought not capitalised for consistency please and Alexithymia component extracted from a rotated principal component analysis, highlighting the need to consider co-occurring symptoms. Together, our findings show that people learn to vary task-switching behaviour as a function of reinforcement, which may be hard or slow to unlearn, as well as interindividual variations along the flexibility-stability trade-off that show a relation to autism.

## Introduction

As humans, we possess the vital ability to flexibly switch between tasks, such as having a phone call while following a recipe. Engaging in task-switching is attractive as it minimises opportunity costs (Kurzban et al., 2013), but also daunting as it requires cognitive control to coordinate both task representations and avoid interference between them (Monsell, 2003; Musslick & Cohen, 2021). Such dilemmas give rise to an additional challenge, which is to learn when to be flexible and when to be stable at the ‘meta-control’ level (see e.g., Braem & Egner, 2018; Dreisbach & Fröber, 2019; Goschke, 2000; Goschke & Bolte, 2014; Musslick & Cohen, 2021). An important research endeavour is to determine the underlying factors driving this regulation of flexibility versus stability, which has important theoretical and clinical implications.

Previous studies demonstrated that people can show meaningful shifts in cognitive flexibility (Braem & Egner, 2018; Dreisbach & Mendl, 2024), depending on environmental requirements in the switch frequency (e.g., Fröber & Dreisbach, 2017; Xu et al., 2024), demand avoidance (Brosowsky & Egner, 2021), time costs (Mendl et al., 2024; Mittelstädt, Miller, et al., 2018), or reinforcement history (Braem, 2017; Held, Vermeylen, Dignath, et al., 2024; Held, Vermeylen, Krebs, et al., 2024). Similarly, on an interindividual level, it has been argued that cognitive flexibility could be a core process contributing to psychopathologies characterised by imbalanced or rigid control strategies (Colzato et al., 2022; Goschke, 2014; Herd et al., 2014). We were interested in whether autism spectrum disorder (from here onward referred to as ‘autism’),^1^ a disorder associated with a strong preference for sameness and repetitive or rigid behaviour (American Psychiatric Association, 2013) is linked to maladaptive regulation of cognitive control strategies, especially at the level of intentional task choice (Poljac & Bekkering, 2012). In addition, we also looked at typically developing individuals scoring high on autism traits, as indicated by their score on autism questionnaires, and other transdiagnostic symptom dimensions. We wanted to test both the effect of an autism diagnosis and elevated autism traits in undiagnosed people, as it is unclear how findings from the subclinical population translate to people diagnosed with (Frazier et al., 2023; Sasson & Bottema-Beutel, 2022). Thus, comparing clinical autism and subclinical traits can provide important nuance to assumed quantitative (i.e., differences in degree along a continuum) compared to qualitative (i.e., differences in kind) differences. While individuals with an autism diagnosis naturally have high levels of autistic traits, we will make a semantic distinction between a group analysis (autism diagnosis versus no diagnosis) and a trait analysis (continuous score of autism symptoms as they are naturally distributed in a comparison group without diagnosis) for this paper.

### Learning of Cognitive Flexibility and Stability-Flexibility Trade-Offs

Fitting with recent frameworks that emphasise control processes can be learned (Abrahamse et al., 2016; Braem et al., 2024; Braem & Egner, 2018; Chiu & Egner, 2019; Doebel, 2020; Logan, 1988; Verbruggen et al., 2014), and theories that emphasise the balancing between the costs and benefits of control (Cools, 2016; Kool, Shenhav, et al., 2017; Shenhav et al., 2013), a cost-benefit framework also holds true for task-switching. Namely, recent studies have demonstrated that when confronted with the option to choose which task to perform next, people will consider the costs (Brosowsky & Egner, 2021; Mendl et al., 2024; Mittelstädt, Miller, et al., 2018) and benefits (Braem, 2017; Held, Vermeylen, Dignath, et al., 2024; Held, Vermeylen, Krebs, et al., 2024) of task-switching. Specifically, Braem (2017) showed that selectively reinforcing task-switching (repeating) in blocks where people were cued which task to do next led to significantly more (less) voluntary task-switching in subsequent blocks where people were free to choose the next task, suggesting people only switched when the benefits (rewards) outweighed the costs.

Interestingly, in addition to testing voluntary task-switching as the intention to be cognitively flexible, this task-switching design also allows the study of measures of the efficiency with which people show cognitive flexibility. When looking at task performance itself, one can study switch costs and task-rule interference effects. Switch costs refer to the observation that performance is more costly (i.e., slower or less accurate) when people have to perform a different task than the one on the previous trial, as opposed to repeating a task (Kiesel et al., 2010; Monsell, 2003). Smaller switch costs are often seen as a measure of greater cognitive flexibility. Task-rule interference effects are characterised as a slower or more erroneous application of the currently relevant task rule (e.g., having to press “left” to categorise stimulus X in task A) when its response is incompatible with the alternative task’s response rule (e.g., stimulus X would require pressing “right” in task B; e.g., Braem et al., 2019; Brown et al., 2007; Held, Vermeylen, Dignath, et al., 2024; Meiran & Kessler, 2008). Smaller interference effects are often seen as a measure of more cognitive stability, or less cognitive flexibility, as the more flexible one’s cognitive system is, the more coactive one tries to keep task representations, at the cost of worse shielding of those representations against one another, i.e., causing more task-rule interference (Armbruster et al., 2012). Notably, reduced interference can also arise from more efficient attentional control or higher processing capacity (e.g., Botvinick et al., 2001; Kane & Engle, 2003; Redick & Engle, 2011), which we view as alternative yet not mutually exclusive accounts.

### Cognitive Flexibility in Autism

A body of literature suggests that autism is associated with deficits in executive functions and cognitive flexibility specifically (e.g., Hill, 2004; Lai et al., 2017; Ozonoff et al., 1991; St. John et al., 2022; Xie et al., 2020). However, other studies on autism have demonstrated that findings on cognitive flexibility strongly vary in magnitude and are largely inconsistent (Geurts et al., 2009; Kenworthy et al., 2008; Leung & Zakzanis, 2014). Interestingly, Geurts and colleagues (2009) highlight an important discrepancy or ‘paradox’ between behavioural inflexibility commonly reported in the everyday functioning of people with autism versus cognitive experimental measures or differences in tasks used to capture cognitive flexibility. For example, a person with autism showing apparently inflexible adherence to specific, nonfunctional routines or rituals might not necessarily perform worse in task-switching or other paradigms presumably measuring cognitive flexibility. One task which has shown a more consistent link with autism is the Wisconsin Card Sorting Task (Berg, 1948), which, however, has been criticised for confounding flexibility with other executive functions and, in comparison to other cognitive flexibility tasks, does not provide explicit task instructions (Geurts et al., 2009; Van Eylen et al., 2011, 2015). Similarly, a recent review paper has pointed out that factors such as additional working memory demand, ambiguity of feedback, and absence of a clear rule to be followed are driving factors of group differences in most reward-based learning paradigms (Van Der Plas et al., 2023). In other words, it may not be cognitive flexibility per se that differs in autism, but rather the learning to adapt control in the absence of clearly defined rules or instructions (see also Poljac et al., 2010; White, 2013). This idea further fits within recent studies suggesting that participants with autism adjust task parameters less as a function of context than neurotypicals (see e.g., Bodner et al., 2019; Fujino et al., 2019; Goris et al., 2018; Palmer et al., 2015), or that they are less likely to change their priors in light of alternating circumstances (De Martino et al., 2008; Farmer et al., 2017; Van Der Plas et al., 2023). As a consequence, they may be less quick to update control settings in response to changing reward contingencies. Below, we will outline how we tested this idea in the current study.

### The Current Study

In this study, we set out to test the effect of selective reinforcement of task alternations (versus repetitions) on voluntary task-switching and task performance-based measures of cognitive flexibility (switch costs and task-rule interference effects). This part is an exact replication of Braem (2017), but using a within-subject study design where each participant underwent both reward conditions (reinforcing task alternations versus repetitions more) and a significantly larger sample size. Additionally, we investigated whether people diagnosed with autism or elevated self-reported autism traits would show less reward-modulated changes in cognitive flexibility. We hypothesised and preregistered that first, people would show increased voluntary task-switching behaviour in an unrewarded voluntary choice phase when rewarded more for task alternations in a preceding cued choice phase, but that autism (diagnosis and high traits) would be related to less reward-modulated changes in cognitive flexibility, i.e., a smaller difference in voluntary task-switching between both reward conditions. Second, Braem (2017) also showed larger task-rule interference effects (less focus) in the group that was rewarded more following task switches. Therefore, we also predicted smaller task-rule interference (henceforth ‘interference’) effects, i.e., relative differences in task performance on stimuli requiring the same vs. different response in the two conflicting tasks, in switch-reinforced blocks, in line with Braem (2017). Consistent with the idea that participants with autism or scoring higher on autism traits would show fewer reward-motivated modulations of cognitive flexibility, we predicted that they would show smaller differences in task-rule interference between the switch-reinforced and repetition-reinforced blocks compared to participants with fewer autism traits or a comparison group. Third, we predicted that people scoring higher on self-reported cognitive flexibility would show larger effects of the switch-dependent reward schedules, as we hypothesised that people scoring high on flexibility also present increased flexibility at the meta-level.

In addition to these directed hypotheses, we also preregistered a more exploratory analysis, i.e., to conduct a principal component analysis (PCA) on a range of clinical questionnaires to see whether the modulation of voluntary task-switching or task performance as a function of reinforcement would be an interesting transdiagnostic marker (similar to the approach deployed by Gillan et al., 2016; Patzelt et al., 2019; Wise et al., 2023). The underlying idea of this approach is that traditional diagnostic categories often share underlying symptom dimensions. If we want to study the mechanisms underlying these shared symptoms, we have to first extract these latent factors and then link them to a specific cognitive process of interest, in our case, reward-based control modulations.

## Methods

The study was approved by the Ethics Committee of the Faculty of Psychological and Pedagogical Sciences of Ghent University (reference number: 2022/081). It was part of a larger multi-day study in which participants underwent five different computer tasks across three sessions within one week. For more details about the context of this study, see below and our overarching preregistration (https://osf.io/9u8gd) and study-specific preregistration (https://osf.io/kp65q). For brevity, preregistered analyses on transdiagnostic traits are reported in Appendix A, with a summary of the main results reported in the Results section. All data and analysis scripts of this study are available on OSF (Held et al., 2022). All participants gave their informed consent for participation and publication.

### Participants

All participants were recruited via the online recruitment system Prolific and were paid £6/hour for their participation (per Prolific guidelines). They received payment after completion of all three sessions within one week. For the present task, they could additionally receive a bonus payment of a maximum of 2.90 pounds. Eligibility criteria for participants were: English as a first language, country of residence either US or UK to limit heterogeneity in the sample, age between 18–35 years old, Prolific acceptance rate of at least 95% and having reported in the Prolific pre-screening survey to have received a diagnosis of autism as a child or adult (preregistered target n = 100) or not have been diagnosed with autism (preregistered target n = 500). The target sample size for this study was based on frequentist power calculations aimed at detecting correlations of *r* = .15 or higher (α = 0.05, two-sided) with a power of 80% (β = 0.2, Lachin, 1981) (n = 350) and adding additional participants within financial constraints. Our power analysis deviates from our Bayesian analyses in the current manuscript, as it was conducted for the entire overarching study comprising multiple projects with different analysis methods (see link above for an overview).

In total, 695 participants completed the task as part of the multi-day study. After preprocessing, the sample for the group analysis (comprising the comparison and autism group) consisted of 418 participants with 67 participants in the autism group (Mean age = 26.39 (5.03) years; 23 women, 34 men, 10 other) and 351 participants in the comparison group (Mean age = 29.15 (4.44) years; 179 women, 169 men, 3 Other). Of these participants from the comparison group, six already participated in an initial pilot experiment (n = 40; due to an error in excluding them from participation) that did not differ in any way from the current study. To avoid data loss, we decided to include their data from the pilot combined with the questionnaire data from the multi-day study, but to exclude them from analyses relying on priors based on pilot data (i.e., voluntary task-switching but not performance analyses). Additional information regarding the socioeconomic status of the sample is depicted in Appendix B. Autism questionnaire scores and factor loadings per group are shown in Table 1. The sample for the autism trait analyses (comprising only comparison participants) consisted of 361 participants (181 women, 176 men, 4 non-binary or undisclosed participants) with complete (questionnaire) data, which is 10 more people than the comparison group because participants with autism trait scores above the criterion were excluded for the group analyses, but included for the trait analyses.

**Table 1:** Autism Scores per Group.


	MEAN (SD)		CRONBACH’S ALPHA (95%-CI)

	AUTISM	COMPARISON	

**Principal component**	2.04 (1.04)	–0.44 (1.01)	

**CATI**	164.98 (23.60)	112.57 (25.02)	0.95 [0.95,0.96]

**AQ**	149.39 (15.09)	114.39 (13.83)	0.91 [0.89,0.92]


*Note*. As the principal component negatively correlated with the questionnaire scores, we here multiplied the means and correlation coefficients by –1 for a more intuitive mapping (higher: more symptoms). CATI and AQ were significantly correlated (*r*(395) = 0.86, *p* < .001) and Cronbach’s alpha of each questionnaire suggests excellent consistency in our sample. AQ: Autism Spectrum Quotient. CATI: Comprehensive Autistic Trait Inventory.

### Preprocessing

Preprocessing was performed both at the level of the entire multiday study and the present experiment. All preprocessing steps were preregistered and inspired by earlier studies with similar methodologies (Braem, 2017; Held, Vermeylen, Dignath, et al., 2024). At the study level, participants were excluded if they did not partake in all three sessions and if they showed three or more ‘task failures’ which were predefined per experiment (e.g., a large number of missing responses, implausible answers or failures on attention checks). This led to the exclusion of 14 participants from the 695 participants who completed the task. In the main analyses, we additionally had to exclude 30 participants due to participation in the pilot experiment or one other earlier study (note that of those, 6 pilot participants were included again for the performance analyses, see above). At the experiment level, we excluded participants with overall accuracies below 65% per task (n = 38) or switch rates below 20% (n = 144). We also removed participants with more than 20 (12%) missing responses on free choice trials (n = 4). This last step was not preregistered but seemed appropriate when checking for carefree behaviour in the scope of quality checks. Next, per participant, we excluded all trials resulting in an error (for the voluntary task-switching and reaction time (RT) analysis) and trials following an error (for the voluntary task-switching, accuracy and RT analyses). We also removed all trials in which the RT was 1.5 times the interquartile range (IQR) above the 75th percentile, and all trials 1.5 times the IQR below the 25th percentile (within-subject), as well as RT faster than 200 ms for the RT and voluntary task-switching analyses. For every analysis, we excluded the first trials.

For the group-level analysis in particular, comparing people with and without an autism diagnosis, we removed all participants who reported not having autism despite having reported so in the pre-screening^2^ (n = 9) and all participants in the neurotypical group who reported having been diagnosed with autism despite not having registered this in the pre-screening (n = 0). Second, participants in the neurotypical group with AQ scores above the cut-off of 32 for ‘autism spectrum disorder’ (or missing values thereof; n = 37) as well as participants in the autism group with AQ scores below the cut-off of 26 for ‘mild autism spectrum disorder’ (or missing values thereof; n = 7), were excluded from the group analyses, as preregistered and standard in many autism patient studies (e.g., Baron-Cohen et al., 2001; Goetmaeckers et al., 2025; Goris et al., 2018, 2022; Woodbury-Smith et al., 2005).

Finally, for the trait analyses, we excluded participants who indicated having autism in the self-report despite not being registered with a diagnosis on Prolific, but kept participants above the cut-off. For all questionnaires used, participants with at least three failures on attention check items were excluded from the respective questionnaire analyses. These items were included to test if participants paid attention to the questionnaires, e.g., by asking them to select a certain response option (Oppenheimer et al., 2009). Categorical predictors were coded using sum-to-zero contrasts, and continuous predictors were scaled.

### Stimuli and Materials

All materials were similar to those reported in Braem (2017), with the main difference being that all word stimuli, adapted from Schneider (2015), were presented in English (see also Held, Vermeylen, Dignath, et al., 2024) and that the experiment was programmed in JsPsych (de Leeuw, 2015). Experimental stimuli consisted of 320 unique target words that can be categorised according to their size (smaller or larger than a basketball) or animacy (animate or inanimate). They were assigned to cued and free choice blocks, where participants were either cued which task to perform or where they could freely choose one, as if they were flipping a coin, respectively (Arrington & Logan, 2004). Both dimensions were crossed orthogonally, resulting in four lists of each 80 words (i.e., animate/small, animate/large, inanimate/small, and inanimate/large). The list was further counter-balanced across blocks and phases so that each word condition appeared equally often. Within the cued phase, these conditions were evenly split between task-repeat and task-switch trials (five trials each per condition). Trial order was fully randomised for each participant, ensuring an unpredictable sequence with a 50% switch rate per cued block. Finally, specific items were randomly drawn from the stimulus lists for each participant to create unique item sequences. Each word was presented in white (30px Verdana) on a black background below a task cue (positioned at 5% of the distance between the centre and the upper or lower border of the screen, respectively). Task cues on cued trials consisted of either vowels (‘A’, ‘E’, ‘I’, ‘O’, or ‘U’) or the consonants ‘V’, ‘F’, ‘L’, ‘Q’, or ‘C’ and they never reoccurred in three subsequent trials to avoid a salient association of specific cue sequences with rewards. The mapping of letter type (vowel or consonant) to task (size or animacy) was counter-balanced across participants. Task cues on free choice trials consisted of a hashtag. Stimuli had to be categorised with the left hand (keyboard buttons ‘S’ and ‘D’) for one task and with the right hand (‘K’ and ‘L’) for the other task. Which task was assigned to which hand was randomised. The left keyboard button always indicated ‘small’ and the right button ‘large’ in the size task. However, the button-to-category assignment in the animacy task (i.e., animate or inanimate) was randomised across participants to ensure counter-balancing of interference. High (‘+ 10’) and low (‘+ 01’) reward feedback of the cued phase was presented in the centre of the screen. No feedback was shown in the free choice phase. Error feedback in the practice phase consisted of the presentation of either ‘Correct!’ or ‘Wrong!’ centrally on the screen. We additionally asked participants an open and closed format question on contingency awareness^3^ and administered the International Cognitive Ability Resource – 16 item version (ICAR; James et al., 2018). This measure consists of subtests Matrix Reasoning, Three-Dimensional Rotation, Verbal Reasoning and Letter and Number Series and has been used before in online autism research (Farmer et al., 2017; Wilson & Bishop, 2020). Relevant to this study, we further administered the Autism Spectrum Quotient (AQ; Baron-Cohen et al., 2001), the Comprehensive Autistic Trait Inventory (CATI, English et al., 2021) and the Cognitive Flexibility Inventory (Dennis & Vander Wal, 2015). The AQ is a well-established and widely used questionnaire in autism research, which further provides clinical cutoff values (Baron-Cohen et al., 2001). The CATI represents a more recent scale which builds on the strengths of the AQ and is particularly suitable for capturing various trait dimensions, for instance, through the addition of dedicated subscales for social camouflage and sensory sensitivity (English et al., 2021). All additional questionnaires are listed in the preregistration (see above), and all (clinical) ones analysed in this paper are listed in Appendix A.

### Procedure

**Figure 1:**
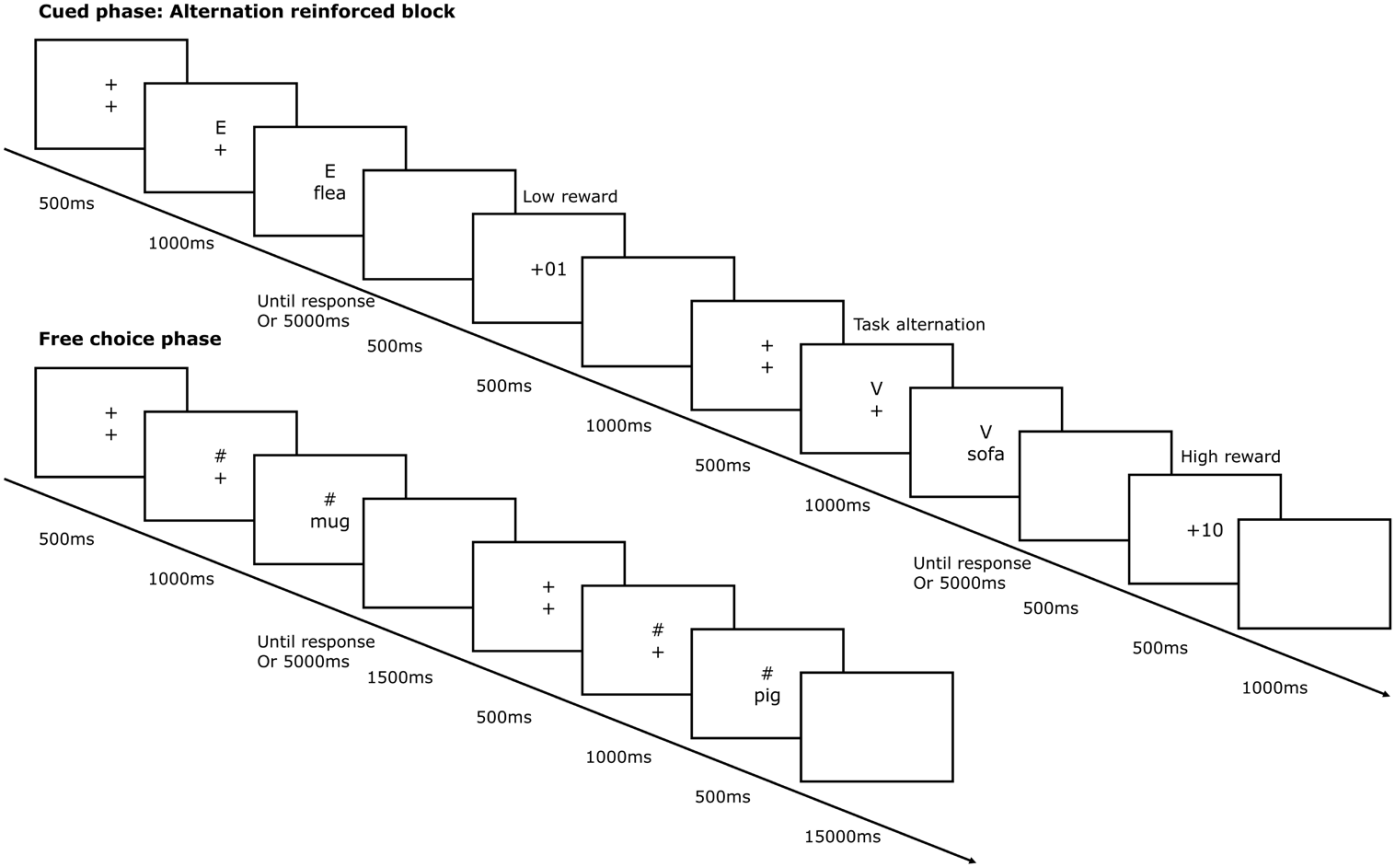
Task Procedure. *Note*. Participants underwent four experimental blocks in total, of which the first half (n = 40 trials) consisted of cued tasks, as indicated by a vowel or consonant cue, and the second half of free choice trials, as indicated by the hashtag. Participants either had to decide whether a word was animate or inanimate, or smaller or larger than a basketball. If correct, participants received points (low/high) based on their reward condition (switch/repeat reinforced) in the cued phase but not in the free choice phase. Reward contingencies switched after the first experiment half. Letters and words were presented in white on a black background in the original experiment.

The current task took about 30 minutes to complete and was part of the second session of a multi-day study (with a total duration of about 1.5 hours), in which participants underwent two additional tasks as well as the ICAR in randomised order. All questionnaires were completed in a third testing session on a later day. At the start of the experiment, participants were instructed about the letter-type-to-task mapping and told about the task and the bonus payment. The experiment started with a practice phase in which they had to categorise four words (one of each category, which were not part of the above-mentioned list) according to their size or animacy. They were instructed that animate refers to any kind of organism, such as animals, plants, nuts, fruits, and vegetables. For the full instructions, see Appendix C. In these practice trials, participants did not receive reward feedback. Each word was repeated three times for a total of 12 practice trials in random order. If participants scored below 75% accuracy in the practice phase (fewer than 9 of 12 practice trials correct), the practice phase started again until participants reached the criterion of 75% accuracy. After successful completion of the practice phase, participants performed four experimental blocks with 80 trials each, out of which the first 40 were cued, and the last 40 were free choice trials. They were instructed that correct responses in the cued phase would be randomly followed by a high or low reward. In reality, participants were rewarded more for either task repetitions or task switches in each block. In the first two blocks, participants were rewarded more for either repetitions or switches (counter-balanced over participants) and in the last two blocks, they were rewarded more for the other task transition type. In switch-reinforced blocks, 80% of switch trials could yield a high reward (‘+10’), while only 20% of switch trials yielded a low reward (‘+01’). For repetition trials in the switch-reinforced blocks, 80% of trials yielded a low reward, and 20% of trials a high reward. This was reversed in repetition-reinforced blocks. In the cued phase, each trial started with two fixation crosses of 500 ms duration with similar spacing as described in the Materials section (see Figure 1). Next, the task cue was presented instead of the upper fixation cross for 1000 ms. Next, the lower fixation cross was replaced by the target word, which stayed on the screen, together with the task cue, until a response was given or for 5000 ms. This was followed by a response-feedback interval of 500 ms (empty screen). The feedback screen in the cued choice phase was presented for 500 ms and consisted of either the reward indication following correct trials or an empty screen following incorrect trials. The intertrial interval was 1000 ms long. In the voluntary phase, the timing was identical except for the reward screen being omitted, resulting in a slightly shorter intertrial interval of 1500 ms.

### Data Analysis

All main statistical analyses were conducted in R (Posit team, 2025) based on Bayesian mixed-effects models using brms (Bürkner, 2018). These analyses were performed, first, on the whole sample including Autism group vs. Comparison group as a two-level factor (group analysis). Second, we also reported the effects of a model on the comparison group alone, to investigate the effect of autism traits by including traits as a continuous predictor (trait analysis). In our voluntary task-switching model, we regressed Reward condition, and its interaction with Autism (Group in the group analysis or Trait score in the trait analysis), Reward order (which reward condition was reinforced first), Age, ICAR scores and Gender onto the participants’ trial-by-trial choices to switch or repeat tasks (voluntary task-switching, modelled with Bernoulli distributions (logit link)). These choices were inferred based on their sequences of choosing a response button from the animacy or size task set, allowing us to infer which task they chose, regardless of the response being correct or incorrect. In the trait analyses, one participant who indicated ‘Prefer not to say’ regarding their gender was combined with participants in the ‘Other’ category (n = 3) due to the small group size. We included random subject intercepts with random slopes for Reward condition per subject. As preregistered, in our main voluntary task-switching model, we used a prior for Reward condition based on initial pilot data from 40 participants, resulting in a normal distribution with a mean of –0.102 and a standard deviation of 0.058. The model was as follows


Voluntary task switch ~ Reward condition * (Group or Autism Traits+Order+Age+IQ+Gender)                              +(1+Reward condition | Subject),

with Group representing Autism or Comparison group in the Group analysis, and Autism Traits representing comparison participants’ score on the first principal component based on an unrotated PCA on the two autism questionnaires (AQ and CATI) in the trait analysis using the *prcomp* function of the stats package (R Core Team, 2021). Questionnaire scores were scaled to have unit variance before analysis. More information on the questionnaires (including Cronbach’s alpha) is depicted in Table 1.

In our task performance models, fitted separately per cued and free choice phase, we predicted RT and accuracy, further adding Transition (switch/repetition), Interference (interfering/non-interfering), and Task (size/animacy) as well as interactions according to the below-specified model.^4^ Interference was coded as a word requiring the same or different response side in the other task (linked to the other hand). Task was added to account for differences in difficulty between the size and animacy task (Held, Vermeylen, Krebs, et al., 2024). For Transition, a trial was defined as a switch if the previous task was different from the current, and a repetition if the task was the same (see Figure 3). Split-half reliabilities for switch costs and interference effects are displayed in Appendix D. They provide an estimate of the reliability of our contrasts, i.e., the mean difference scores per individual between switch versus repetition trials (switch costs) and interfering versus non-interfering trials (interference costs). Descriptive data regarding independent and dependent variables of the group analyses are presented in Appendix E. We added random slopes for the within-subject main effects and random subject intercepts, as well as random slopes for Task per stimulus (word) and random stimulus intercepts to account for the intrinsic variability across different category words, and ensure our effects are not driven by idiosyncratic features of our specific stimuli. RT was modelled using a shifted lognormal distribution and accuracy with a Bernoulli distribution (logit link):


$$\begin{array}{cc} \text{Task performance} & \sim \;{\left(\text{Reward condition}+\text{Transition}+\text{Interference}\right)}^{2}\\ & *\;\left(\text{Group or Autism Traits}+\text{Order}+\text{Task}+\text{Age}+\text{IQ}+\text{Gender}\right)\\ & +\;(1+\text{Reward condition}+\text{Transition}+\text{Interference}+\text{Task}\;|\;\text{Subject})\\ & +\;(1+\text{Task}\;|\;\text{Stimulus}). \end{array}$$

To infer statistical significance, we preregistered to report the posterior using a 90% highest posterior density interval around the mode for directed hypotheses (in the predicted direction) and 95% highest posterior density credible intervals for undirected hypotheses, checking whether these include zero or not. We additionally preregistered to report posterior probability values (ps), which are defined as the proportion of the posterior parameter distributions that are above or below 0 (reported here depending on the *predicted* direction of the effect). We do so for preregistered hypotheses only. Interactions were interpreted based on conditional effects plots, except effects involving gender, autism group or traits, where we additionally consulted post hoc tests using the emmeans package (Lenth, 2022). For all models, we made sure that there were no error or warning messages, that the chains converged and that all R-hats were between 0.99 and 1.01. For the group models, comprising most of the trait analysis sample, and where it is most critical due to the fairly small autism group, we additionally checked posterior predictive checks, i.e., how well the predicted values fit the observed values, whether correlations between predictors at each draw were below 0.8 (collinearity), and whether the pareto k values were below 0.7 (influential cases).

## Results

We first studied the effects of our reward manipulation and autism (group and traits) on voluntary task-switching behaviour, as a more intentional marker of cognitive flexibility (i.e., the intention to switch). Second, we discuss the effects of our reward manipulation on task performance (i.e., reaction times and accuracy), where we focused on (modulations of) task-rule interference effects, which are taken to reflect the efficiency of task-switching behaviour or cognitive flexibility. Again, we also studied this in the context of having an autism diagnosis or scoring high on autism. Per voluntary task-switching and performance analysis, we will initially report preregistered analyses, followed by other significant effects based on the larger group analysis, except where they involve autism traits, in which case results stem from the trait analyses. In general, we will report interactions of interest with autism group or traits but not with the demographic variables, which mainly served to eliminate potential confounds in the model, i.e., that interactions might be attributed to age, gender, or ICAR. Third, we will report our interindividual difference analyses, i.e., on self-reported cognitive flexibility and the principal components extracted from our principal component analysis as described in the respective section. Again, we will focus on the interactions or main effects involving these variables. Complete results tables can be found in our Appendices as indicated per the reported model. For control variables, we only report main effects but no interactions with experimental variables, as these were not of main interest to our study.

### Voluntary Task-Switching

**Preregistered effects.** Our voluntary task-switching group model (n = 412, due to the exclusion of participants participating in the pilot study) revealed no main effect of Reward condition (*b* = –0.007, 90%-CI [–0.054,0.038], ps_>0_ = 0.401), indicating that participants did not switch (repeat) more in the switch (repeat) reinforced blocks. This effect did not interact with Group (*b* = –0.011, 90%-CI [–0.047,0.025], ps_<0_ = 0.688), indicating that there was no modulation of the effect of Reward condition by Group, and there was no main effect of Group (*b* = 0.085, 95%-CI [–0.028,0.197]), suggesting people with autism did not show more or less voluntary task-switching.

In the trait analyses in our comparison group, we also found no main effect of Reward condition (*b* = –0.041, 90%-CI [–0.105,0.023], ps_>0_ = 0.149), and no interaction between Reward condition and Autism traits (*b* = –0.004, 90%-CI [–0.028,0.019], ps_>0_ = 0.376), nor a main effect of Autism traits (*b* = –0.018, 95%-CI [0.093,0.058]), indicating that there was no main effect thereof on voluntary task-switching or its hypothesized modulation by Reward condition (see Appendix F for all voluntary task-switching analyses).

**Other effects and follow-up analyses.** Interestingly, the only predictors of voluntary task-switching in the group model were Reward order (*b* = –0.230, 95%-CI [–0.388,–0.069]) and ICAR (*b* = 0.203, 95%-CI [0.124,0.281]), with participants switching more when they were rewarded more on switches first, and when they were scoring higher on cognitive abilities. None of the other main effects or interactions reached significance.

**Figure 2:**
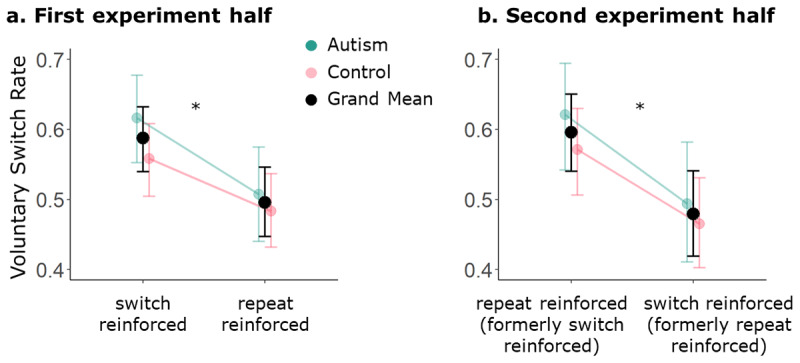
Voluntary Switch Rate in The First and Second Experiment Half. *Note*. The model estimated main effect of voluntary task-switching across groups (autism or comparison group) per first **(a)** and second **(b)** experiment half. Our results suggest that, across groups, participants rewarded more on switches in the first experiment half alternate more between tasks in the first experiment half, but they do not seem to unlearn this mapping after the contingency reversal in the second half. The green, pink and black lines represent the marginal means extracted from emmeans (Lenth, 2022) averaged across gender and the means of age and ICAR.

As described under our preregistered effects, we did not find a main effect of Reward condition, which we reasoned could be related to the within-subject design of the current study. Namely, the contingency reversal in the middle of the experiment may have disrupted potential learning, as also suggested by the main effect of Reward order described above. Since the order of reward conditions was counter-balanced, we could thus treat this first half as a between-subject design with the first encountered reward condition as the between-subject factor. Therefore, and because Braem (2017) already observed an effect of Reward condition in the first block, we decided to also analyse the first experiment half separately. These analyses affirmed the expected main effect of Reward order (*b* = –0.192, 95%-CI [–0.343,–0.041], ps_>0_ = 0.008), i.e., the between-subject equivalent of Reward condition in this analysis, with participants rewarded more on switches switching more than participants rewarded more on repetitions (see Figure 2).

### Task Performance Measures of Cognitive Flexibility

Next, we turned to the task performance data (reaction times and accuracy). While we report the performance data for both free choice and cued task trials, to which models were fitted separately, caution is warranted when interpreting reaction times and accuracy in free choice trials, as performance efficiency is inherently influenced (or ‘confounded’) by the task participants choose to perform.

**Preregistered effects.** Our reaction time group results did not confirm our preregistered hypothesis of increased task-rule interference effects in switch-reinforced blocks, based on Braem’s finding (2017). Using our preregistered one-sided test (90% credible interval), both models did not show an interaction of Reward condition and Interference in the expected direction, i.e., participants did not show larger interference effects in switch-reinforced blocks as compared to repeat-reinforced blocks, in free (*b* = –0.006, 90%-CI [–0.010,–0.001], ps_<0_ = 0.986) or cued choice trials (*b* = –0.007, 90%-CI [–0.013,–0.000], ps_<0_ = 0.953). This was also not further modulated by Group (autism versus comparison) in either (free or cued) trial type (*b* = –0.003, 90%-CI [–0.006,0.000], ps_>0_ = 0.082; *b* = 0.002, 90%-CI [–0.002,0.007], ps_>0_ = 0.772). In the trait reaction time analysis studying the effect of autism traits in the comparison group, we again found no evidence for an interaction between the Interference by Reward condition interaction and Autism traits (*b* = –0.000, 90%-CI [–0.002,0.002], ps_<0_ = 0.544; *b* = –0.001, 90%-CI [–0.004,0.001], ps_<0_ = 0.765), suggesting no differences in reward-motivated changes in cognitive flexibility versus stability.

In our accuracy group model, we again did not observe a significant Reward condition by Interference interaction in the free (*b* = 0.039; 90%-CI [–0.031,0.109], ps_>0_ = 0.824), or cued (*b* = –0.013, 90%-CI [–0.066,0.040], ps_>0_ = 0.339) choice trials, in line with Braem (2017). We also found no modulation of the Reward condition by Interference interaction by Group (*b* = 0.025, 90%-CI [–0.023,0.074], ps_<0_ = 0.199; *b* = –0.011, 90%-CI [–0.048,0.026], ps_<0_ = 0.685). Similarly, there was no significant interaction between Reward condition, Interference, and Autism traits (*b* = 0.030, 90%-CI [–0.001,0.061], ps_>0_ = 0.942; *b* = –0.008, 90%-CI [–0.033,0.015], ps_>0_ = 0.286) in the trait analysis.

**Figure 3:**
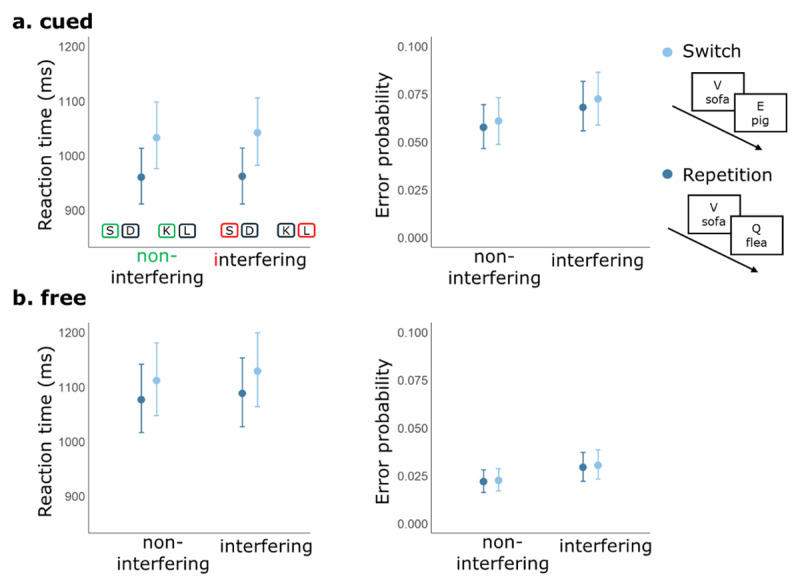
Switch Costs and Interference Effect in Cued and Free Choice Trials. *Note*. The model-estimated reaction time and accuracy interference effect and switch costs per **(a)** cued and **(b)** free choice experiment phase. Participants were slower on switch trials compared to repeat trials and less accurate on non-interfering compared to interfering trials. Next to and below the graphs is depicted what constitutes a repeat versus switch trial and a non-interfering versus interfering keyboard response.

**Other main effects unrelated to autism diagnosis or traits.** Regarding other effects in the reaction time group models, we observed overall switch costs as indicated by the main effect of Transition (*b* = –0.020, 95%-CI [–0.029,–0.011], *b* = –0.054, 95%-CI [–0.064,–0.044]), with participants being slower on switch trials (see Figure 3). We also observed a main effect of Task (*b* = –0.020, 95%-CI –0.027,–0.013]; *b* = –0.037, 95%-CI [–0.047,–0.027]), indicating slower responses for the size task. In cued choice trials specifically, we also found a main effect of Reward order (*b* = –0.040, 95%-CI [–0.076,–0.004]), with participants being overall slower when rewarded more on switches first. In both group reaction time analyses (free or cued task phase), we saw a main effect of ICAR (*b* = –0.049, 95 CI [–0.081,–0.017]; *b* = –0.072, 95%-CI [–0.109,–0.034]), with people scoring lower on ICAR being slower in the experiment. In cued trials, we additionally saw a main effect of Gender (*b* = –0.102, 95%-CI [–0.184,–0.019]), with women being significantly faster than men.

In accuracy, we observed a main effect of Interference (*b* = 0.173, 95%-CI [0.059,0.288]; *b* = 0.091, 95%-CI [0.014,0.170]) with participants being more accurate on non-interfering trials (see Figure 3). On cued trials, we also saw a main effect of Task (*b* = 0.078, 95%-CI [0.004,0.154]) with participants being more accurate on the animacy as compared to the size task. In both accuracy models, we observed main effects of Age (*b* = 0.163, 95%-CI [0.052,0.274]; *b* = 0.082, 95%-CI [0.003,0.160]) and ICAR (*b* = 0.325, 95%-CI [0.214,0.436]; *b* = 0.293, 95%-CI [0.213,0.374]), with participants being more accurate with increasing age and higher ICAR scores.

**Other main and interaction effects related to autism diagnosis or traits.** Interestingly, in both reaction time group analyses (free and cued task phase), we saw a main effect of Group (*b* = 0.057, 95%-CI [0.013,0.101]; *b* = 0.069, 95%-CI [0.017,0.122]), with the autism group being slower in the experiment. The reaction time analysis on cued trials further showed a three-way interaction between Reward condition, Transition and Group (*b* = 0.008, 95%-CI [0.002,0.013]). Visual inspection of the marginal effects indicated that, while the comparison group demonstrated a relatively reduced switch cost under switch-reinforced conditions, this effect was inverted in the autism group, where the selective reinforcement of task switches appeared to coincide with larger switch costs. Since there was no clear theoretical interpretation, we deemed this effect fragile and a possible byproduct of the large sample size.

**Figure 4:**
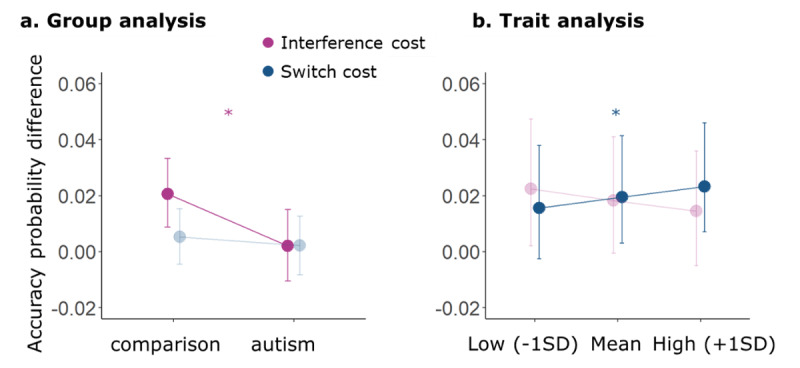
Interference Effects and Switch Costs in Cued Choice Trials in the Group and Trait Analyses. *Note*. While only reaching significance in the interference effect of the group analysis and switch costs in the trait analysis (non-transparent), our results suggest an antagonistic relationship between interference effects (stability) and switch costs (flexibility), mostly in the trait analyses, where people scoring higher on autism traits showed smaller interference effects at the cost of larger switch costs. The blue and purple lines represent the difference scores between the marginal means of repeat versus switch and interfering versus non-interfering trials (as depicted in Figure 3), extracted from emmeans (Lenth, 2022). For visualisation purposes, we show autism traits at the mean, as well as at one standard deviation below and above the mean.

Interestingly, in the accuracy group analyses, even though we did not find the preregistered reduced modulation of interference effects between blocks in autism, we did observe a significant interaction indicating the overall Interference effect differed by Group in the cued phase (*b* = –0.068, 95%-CI [–0.123,–0.013]), with participants in the autism group showing generally smaller interference effects than participants in the comparison group (see Figure 4a), suggestive of more cognitive stability. Moreover, this cued model also showed a Reward condition by Group interaction (*b* = –0.055, 95%-CI [–0.107,–0.003]), with participants with autism showing better performance in switch relative to repeat reinforced blocks as compared to the comparison group. Last, in the cued accuracy group model, we saw a significant three-way interaction of Transition, Interference and Group (*b* = 0.047, 95%-CI [0.003,0.091]). Inspection of the marginal means suggested that the comparison group showed lower performance on switch rather than repeat trials only on interfering trials, whereas this pattern was reversed in the autism group. Interestingly, in the trait accuracy analyses of the cued choice trials, we did observe a significant interaction between Transition and Autism traits (*b* = –0.032, 95%-CI [–0.062,–0.002]), with larger switch costs in people scoring higher on autism traits (see Figure 4b, see Appendix G for the full results tables of all accuracy analyses), suggesting reduced cognitive flexibility in people with elevated autism traits. The analysis of the cued reaction times also showed a significant Reward condition by Autism trait interaction (*b* = –0.008, 95%-CI [–0.015,–0.000]) with participants scoring higher on the autism component being faster on switch reinforced as compared to repeat reinforced blocks (see Appendix H for the full results tables of all reaction time analyses).

Taken together, exploratory performance accuracy results in the cued choice phase suggest enhanced cognitive stability at the cost of reduced cognitive flexibility in the autism group and autism traits. That is, participants with an autism diagnosis showed reduced interference effects in light of stable task switch costs (Figure 4a), and participants with more autistic traits showed almost reduced interference effects in light of increasing task switch costs (Figure 4b). These results were specific to the cued choice phase, which is the phase that allowed us to measure performance efficiency devoid of the confounding effects of task choice (as people could choose which task to perform in the free choice phase).

### Cognitive flexibility and Transdiagnostic Trait Analysis

We also preregistered to run the main (voluntary task-switching) model on the cognitive flexibility inventory (CFI, Dennis & Vander Wal, 2015) to test if participants scoring higher on self-reported cognitive flexibility show a stronger modulation of the reinforcement effect or overall voluntary task-switching. To this end, we also ran our main analyses on voluntary task-switching, including scores on the CFI. These analyses revealed no main effect of CFI scores on voluntary task-switching (*b* = 0.021, 90%-CI [–0.048,0.091], ps_<0_ = 0.305), nor an interaction effect with Reward condition (*b* = 0.008, 90%-CI [–0.017,0.034], ps_<0_ = 0.294). In other words, people scoring higher on self-reported cognitive flexibility were neither more likely to switch, nor more likely to learn and be influenced by our reward manipulation.

In our analysis of transdiagnostic traits, we extracted four main factors, namely ‘Depressive symptoms/Rumination’, ‘Social withdrawal’, ‘Alexithymia’, and ‘Compulsivity and intrusive thought’ (see Appendix A for more detail). However, we found no main or interaction effects between the four principal component loadings in our voluntary task-choice analyses. Our free choice reaction time model revealed an interaction between Transition and the Alexithymia component (*b* = –0.007, 95%-CI [–0.012,–0.001]), indicating larger switch costs with higher alexithymia. In this same model, we also saw a significant three-way interaction between Reward condition, Interference, and Depressive symptoms/ Rumination (*b* = –0.005, 95%-CI [–0.009,–0.001]), suggesting larger interference effects in repetition compared to switch reinforced blocks in people scoring high on this component. It also revealed a three-way interaction between Transition, Interference, and Compulsivity and intrusive thought (*b* = 0.004, 95%-CI [0.001,0.007), suggesting smaller interference effects on repetition compared to switch trials in people scoring high on this component. In the cued reaction time analysis, we observed a significant three-way interaction between Reward condition, Transition, and Depressive symptoms/ Rumination (*b* = –0.005, 95%-CI [–0.010,–0.000]), suggesting relatively smaller switch costs in repetition reinforced compared to switch reinforced blocks in people scoring lower on this component.

Both the free and cued accuracy models revealed a significant effect of Social withdrawal (*b* = 0.143, 95%-CI [0.012,0.275]; *b* = 0.111, 95% [0.017,0.205]), with people scoring high on this component performing more accurately. Moreover, the free choice accuracy revealed significant main effects of Alexithymia (*b* = –0.155, 95%-CI [–0.283,–0.028]) and Compulsivity and intrusive thought (*b* = –0.143, 95%-CI [–0.268,–0.020]), with people scoring high on these components performing less accurately. This same model also revealed a three-way interaction between Reward condition, Interference, and Alexithymia (*b* = 0.055, 95%-CI [0.007,0.102]), with people scoring high on this component having larger interference effects in switch reinforced blocks compared to repeat reinforced blocks, and this pattern being reversed for people scoring low on this component. Finally, the cued choice model showed a significant two-way interaction between Transition and Compulsivity and intrusive thought (*b* = 0.043, 95%-CI [0.007,0.080]), suggesting larger switch costs in people scoring higher on this component. In light of this finding (showing a similar pattern to autism traits), and the other significant effects, we were wondering how highly each of these components is correlated with the autism factor. These correlations reveal significant correlations for each of them, as displayed in Table 2.

**Table 2:** Correlations between the autism trait and the four principal components.


COMPONENT	PEARSON’S r	*p*-value

Depressive symptoms/ Rumination	0.449	<.001

Social withdrawal	0.730	<.001

Alexithymia	0.521	<.001

Compulsivity and intrusive thought	0.572	<.001


*Note*. Since the autism component loaded negatively on each of the autism questionnaires, and the principal components positively on their respective items, we multiplied the autism component by –1 before computing correlations.

## Discussion

In this study, we investigated whether people show variations in cognitive flexibility as a function of different reinforcement contingencies, and autism diagnosis or traits. Using a within-subjects design where all participants were subjected to both conditions, in which switch versus repetition trials were reinforced more, we could further study the extent to which they were able to unlearn first-learned contingencies, i.e., to learn new contingencies after the reversal. First, we replicated the observation that selectively reinforcing task switches over task repetitions resulted in subsequently increased voluntary task-switching behaviour. These findings are concordant with theories highlighting the learning perspective on cognitive control (Abrahamse et al., 2016; Braem et al., 2024; Braem & Egner, 2018; Chiu & Egner, 2019; Doebel, 2020; Logan, 1988; Verbruggen et al., 2014) and studies showing that such learning is slow and cannot immediately be unlearned when the contingencies reverse (Braem et al., 2024). However, these modulations in voluntary task-switching were not related to autism diagnosis or traits.

Second, and in contrast to Braem (2017), we did not replicate the observation that reinforcing task switches also resulted in increased task-rule interference effects. It is noteworthy that, while this was also the case in Braem’s (2017) study, interference effects in this task are expected to be rather small, as they were merely linked to the response side per hand, rather than the same finger of the same response hand, which may have limited room for modulations. Interestingly, our observed absence of a modulation is also in line with other recent studies, such as those by Geddert & Egner (2022) or Chiu & Nack (2025), who similarly showed that changing the frequency of task switches does not affect task-rule interference effects, and changing the frequency of task interference does not affect switch costs. Another recent study on auditory attention switching found that manipulating the proportion of switch trials affected switch costs but not interference effects, while manipulating the proportion of interfering trials affected interference effects but not switch costs (Strivens et al., 2024). While manipulating the frequency of switches is not the same as reinforcing them, the underlying idea is similar: learning about suitable control settings in one condition (frequency or reward) does not antagonistically affect the other. This indicates that people can independently adjust the way they approach task-switching and task-rule interference conditions, and suggests that we should see these as two largely independent processing styles and stages (Egner, 2023; Geddert et al., 2025; Nack & Chiu, 2024), or that people can also dynamically navigate a flexibility-stability trade-off between competing task representations during these sequential stages of task and response selection (Dreisbach et al., 2024; Ritz et al., 2024). Namely, Dreisbach et al. (2024) argued that (in)dependence of stability and flexibility is itself dependent on the level of information processing, and dynamic adjustments of task-switching and interference processing may rely on relatively independent processing stages. Yet, at the same time, both processes are still thought to be ultimately constrained by the same underlying task representations (Musslick & Cohen, 2021). Therefore, an individual’s trait-level ‘stability’ can still manifest as a unified shift across both stages: promoting better task focus (reduced interference) while making switching between representations more difficult (increased switch costs), which also motivated us to study trait-level differences.

In contrast to our hypothesis, reinforcement-specific performance adjustments of cognitive flexibility and stability (with a focus on interference effects) were not modulated by autism diagnosis or autism traits. However, an exploratory finding was that, in the cued reaction time and accuracy analyses, higher autism traits (reaction time) or diagnosis (accuracy) were linked to overall better performance (faster and more accurate) in switch as compared to repeat reinforced blocks. Thus, it seems that particularly participants scoring higher on autism or with an autism diagnosis invested more effort in switch-reinforced blocks where earning rewards was inherently more effortful, which would be in line with a cost-benefit trade-off of control (Kool, Gershman, et al., 2017; Kool & Botvinick, 2013; Shenhav et al., 2013). This pattern further fits with a recent study, which found that participants rewarded more on switches showed a smaller difference in performance between an easier and more difficult task than the group rewarded more on repetitions (Held, Vermeylen, Krebs, et al., 2024). While speculative, it could be that the more pronounced pattern in autism diagnosis and traits is due to (over)compensation in terms of control adjustments, which, outside the laboratory, might help the masking of symptoms (see also similar reasoning that compensatory strategies often mediate between symptoms and cognitive dysfunctions in autism; Brunsdon & Happé, 2014). Future studies are needed to test this idea.

Moreover, while we did not see modulations of interference effects or switch costs due to our reinforcement schedule, exploratory findings revealed general modulations of a flexibility-stability trade-off related to both autism traits and diagnosis in task performance, independent of our reward schedule. Specifically, participants with an autism diagnosis or scoring higher on autism traits showed overall smaller interference effects as compared to the comparison group or people scoring lower on autism traits. Conversely, people in the comparison group who scored higher on autism traits also showed larger switch costs. These findings are in line with one core symptomatology of people with autism, i.e., focusing on single tasks with reduced attention to external stimuli (American Psychiatric Association, 2013). It also fits with a few studies showing reduced cognitive flexibility in autism (South et al., 2007), though these findings are largely inconsistent in the literature (Geurts et al., 2009; Van Der Plas et al., 2023; Van Eylen et al., 2011). This could mean that our paradigm is a more suitable behavioural task to bring to light this core symptomatology of inflexibility often experienced by people with autism, but not captured in other behavioural tasks (see also Introduction and the flexibility paradox). Conversely, it is also noteworthy that while we did not find differences in voluntary task-switching, some previous studies have (Hoofs et al., 2018; Poljac et al., 2017). It would be interesting to investigate what might have driven these effects in our study, e.g., whether they were linked to the lack of clear task instructions, an important feature known to bring out group differences (Lage et al., 2024; Van Der Plas et al., 2023; Van Eylen et al., 2011). In addition to the absence of instructions, a unique aspect of our design was that it was less constrained compared to past research, as it deployed unique stimuli on each trial. It could be possible that designs using recurring stimuli, as preferred by participants with autism who show a strong preference for sameness, might overshadow potential differences in performance.

A third hypothesis was that people scoring higher on self-reported cognitive flexibility would show larger effects on switch-dependent reward schedules, for which we found no evidence. We also preregistered to run analyses to test whether other psychiatric traits are associated with alterations in behaviour, following the approach of a principal component analysis on a vast set of psychiatric questionnaires (similar to Gillan et al., 2016; Wise et al., 2023). These analyses revealed that the pattern of larger switch costs found for autism traits in the cued choice accuracy model was also found for the Compulsivity and intrusive thought dimension, and also for the Alexithymia component in the free choice reaction time model. Both of these components were significantly correlated with autism traits, in line with the literature that autism is often co-occurring with symptoms such as compulsivity, alexithymia and depressive symptoms (Bloch et al., 2021; Ghaziuddin et al., 2002; Kinnaird et al., 2019; Lever & Geurts, 2016; O’Loghlen et al., 2025; Poquérusse et al., 2018). These findings underscore the importance of taking additional symptoms into account.

There are also limitations in our study. First, each block only consisted of forty cued and free choice trials, of which error trials did not yield reward feedback. Thus, learning in the cued phase was limited to even fewer trials, which might have contributed to difficulties with learning or unlearning the reward schedule. Future studies can help determine whether learning from more trials would result in stronger (reversal) learning. Second, we did not have a baseline condition, which makes it impossible to dissect whether our reward manipulation acted more strongly when reinforcing switch or repetition trials, or both. It would be interesting to compare our reinforcement schedules to a control condition where both are equally rewarded. Third, our study relied on ‘coin flip’ instructions to encourage participants to switch tasks between trials (Arrington & Logan, 2004). This analogy is typically used to guarantee enough switches for data analysis, as people tend to show a task repetition bias (e.g., Fröber & Dreisbach, 2017; Held, Vermeylen, Krebs, et al., 2024; Kessler et al., 2009; Liefooghe et al., 2010). However, it has been debated whether such instructions produce genuine voluntary task choices or whether switch behaviour is confounded by the artificial demand to generate random sequences (Mayr & Bell, 2006). When people are instructed to ‘choose randomly’, they are often thought to engage in deliberate random-sequence generation (Baddeley et al., 1998; Reynders et al., 2026), which itself requires executive resources and can interfere with the interpretation of task choice (Mittelstädt, Dignath, et al., 2018). This implies that the observed switching could both reflect cognitive flexibility and compliance with this secondary task instruction, which may have further impacted reward learning and differences in autism (traits). Moreover, the voluntary task phase in our experiment always followed the cued choice phase, which might have produced carry-over effects of the switch rates established in the cued phase (Fröber et al., 2022). These aspects pose an additional challenge in determining whether voluntary task-switching reflected participants’ intrinsic tendencies and adaptation to the reward context, or rather the inertia of switch patterns developed during the cued task. Effects reported here likely result from an interaction between all of the above factors. One solution to tell apart potential confounding effects of randomness instructions and switch inertia in future studies could be the use of self-organised task-switching paradigms (e.g., Mittelstädt, Miller, et al., 2018; Monno et al., 2021), hybrid free–forced choice tasks (Fröber et al., 2022; Fröber & Dreisbach, 2017; Held, Vermeylen, Krebs, et al., 2024), performance-based decision paradigms (Mittelstädt et al., 2019), or free concurrent dual-tasking (Brüning et al., 2021; Reissland & Manzey, 2016). However, such studies will need careful calibration with respect to rewarding abstract control strategies such as task-switching. In fact, a previous study in our lab using a hybrid version of the current study found that participants showed a clear task preference. They concluded that participants may have misattributed the reward to the task rather than the more abstract task-sequence level, and that coin flip instructions might be a suboptimal necessity to have people entertain this level in the first place (Held, Vermeylen, Krebs, et al., 2024). Lastly, our sample was collected online. While this enabled us to test a comparably large sample size, it comes with the limitation of having to trust individuals’ self-reported autism diagnosis and questionnaire scores, rather than relying on formal diagnosis or clinical interviews. However, we took additional precautions in our preprocessing, and differences in means on the autism questionnaires suggested that the groups significantly differed in the expected direction. Another concern might be that performing the task online in a home environment might not have brought out differences that would arise in a lab environment, and people partaking in online experiments likely represent a subgroup that is not necessarily representative of all high-functioning people with autism (and neurotypicals). Future studies should investigate potential differences between different online or offline participant groups.

## Conclusion

In a large study comprising neurotypical participants and participants diagnosed with autism, we demonstrate that the reinforcement of task switches leads to changes in people’s voluntary task-switching behaviour as an intentional measure of cognitive flexibility. Specifically, people showed increased voluntary task-switching in response to this reward contingency, which persisted after the reward contingency changed. However, efficiency measures of cognitive flexibility (i.e., switch costs and interference effects) were not affected by the reward manipulation. Interestingly, while autism diagnosis or traits were not related to changes in voluntary task-switching, people with an autism diagnosis or scoring higher on autism traits seemed to show more general differences in the efficiency measures, namely (1) increased global adaptations to the different reward contexts, and (2) a shifted flexibility-stability trade-off towards better task focus at the cost of less flexibility. Together, we believe our findings point to important modulators and boundary conditions of cognitive flexibility in task-switching and decision-making, and may inspire future studies tapping into the mechanisms underlying the learning of cognitive control in both neurodivergent and neurotypical populations.

## Additional File

The additional file for this article can be found as follows:

10.5334/joc.511.s1 Appendices. Appendix A to H.

## Data Availability

All data and analysis scripts of this study are available on OSF (Held et al., 2022). DOI: 10.17605/OSF.IO/W5X29.

## Footnotes

This paper uses an abbreviated form of the diagnostic term (autism spectrum disorder) and refers to a person with a diagnosis of autism spectrum disorder as a person with autism. This choice does not imply a stance regarding person-first and identity-first language, as there is no current consensus. We acknowledge and respect all language preferences for referring to individuals with a diagnosis of autism spectrum disorder.

After contacting the according participants per message on Prolific, we reincluded those who confirmed their diagnosis.

Note that the groups did not differ in terms of correctly identifying their reward contingency (*b* = –0.15, 95%-CI [–0.62,0.30]).

This model did not lead to good convergence in the free choice accuracy model of the control group as indicated by inspection of the trace plots, which is why we ran it excluding the demographic variables. As the main effects of interest were not significant in this model, we did not continue to perform step-wise inclusion per predictor variable but will interpret interactions with (demographic) variables based on the group model only.
