# Supplementary material for: Balancing Cognitive Flexibility and Stability: The Role of Reward, Autism, and Transdiagnostic Traits
Source: J Cogn. 2026 Jul 24;9(1):38. doi: 10.5334/joc.511 (PMC13398604; doi:10.5334/joc.511)
Supplement: Appendices. — Appendix A to H. [file joc-9-1-511-s1.pdf]

Appendix A

Table A1

Clinical Questionnaires

Questionnaire		
Apathy Evaluation Scale	AES	Marin et al., 1991
Autism-Spectrum Quotient	AQ	Baron-Cohen et al., 2001
Anxiety Sentitivity Index-3	ASI-3	Taylor et al., 2007
ADHD Self-Report Scale (ASRS-v1.1) Symptom Checklist	ASRS	Adler et al., 2012
Comprehensive Autistic Trait Inventory	CATI	English et al., 2021
Problem Gambling Severity Index	CPGI	Ferris & Wynne, 2001
Depression Anxiety & Stress scale	DASS	Lovibond, 1995
Eating Attitudes Test	EAT	Papini et al., 2022
The Liebowitz Social Anxiety Scale	LSAS	Heimberg et al., 1999
Obsessive Compulsive Inventory	OCI-R	Foa et al., 2002
Perth Alexithymia Questionnaire	PAQ	Preece et al., 2018
Rumination scale	RRS	Treynor, 2003
Zung Self-Rating Depression Scale	SDS	Zung, 1965
Short scales for measuring schizotypy	SSMS	Mason et al., 2005
Barratt Impulsiveness Scale	BIS	Patton et al., 1995

Exploring Relations With the Cognitive Flexibility Inventory and Transdiagnostic Traits

Principal Component Analyses on All Questionnaire Items

In addition to our main hypotheses, we also preregistered a more exploratory analysis, i.e., to conduct a principal component analysis (PCA) on a range of clinical questionnaires to see whether the modulation of voluntary task-switching or task performance as a function of reinforcement would be an interesting transdiagnostic marker (similar to the approach deployed by Gillan et al., 2016; Patzelt et al., 2019; Wise et al., 2023). Questionnaires included in this study differed slightly from the ones used by these authors (most importantly, comprising autism questionnaires, for an overview, see Table A1). The PCA was performed based on all comparison participants participating in the larger study with complete item data (n = 475).

By studying the scree plot (see Figure A1), we identified three components. We then performed a rotated principal components analysis using the principal function of the psych package (Revelle, 2007) and using Oblimin rotation. The first component seemed to load most strongly on 'Depressive symptoms/ Rumination', explaining 12% of the total variance. The item most strongly loading most on this component was, e.g., 'I felt I wasn't worth much as a person' (Depression Anxiety & Stress scale, DASS, Lovibond, 1995). The second component explained 9% of the variance and seemed to load on 'Social withdrawal' with the top item being 'Social interaction is easy for me' (CATI). The third component explained 6% of the variance and tapped into 'Alexithymia' with the top item being 'It's strange for me to think about my emotions' (Perth Alexithymia Questionnaire, PAQ, Preece et al., 2018). The fourth component, i.e., the first after

the elbow, seemed to load on 'Compulsivity', with items such as 'I like to arrange items in rows or patterns' (CATI). This component explained 3% of the variance and closely resembles the factor 'Compulsivity and intrusive thought', as identified by Gillan et al. (2016) as the second component. Due to this similarity, we decided to also include it in the analyses to allow comparability of the results. Full results tables of all models fitted on the subset of participants with available components (N = 328) are depicted in Tables A2-A6.

Figure A1

Screeplot

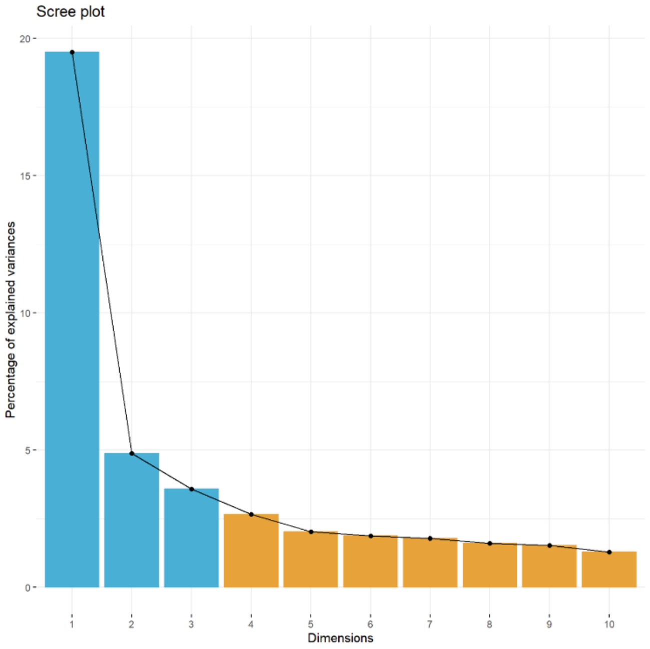

References A1

Adler, L. A., Shaw, D. M., Spencer, T. J., Newcorn, J. H., Hammerness, P., Sitt, D. J., Minerly, C., Davidow, J. V., & Faraone, S. V. (2012). Preliminary Examination of the Reliability and Concurrent Validity of the Attention-Deficit/Hyperactivity Disorder Self-Report Scale v1.1 Symptom Checklist to Rate Symptoms of Attention-Deficit/Hyperactivity Disorder in Adolescents. *Journal of Child and Adolescent Psychopharmacology*, *22*(3), 238–244. https://doi.org/10.1089/cap.2011.0062

Baron-Cohen, S., Wheelwright, S., Skinner, R., Martin, J., & Clubley, E. (2001). The Autism-Spectrum Quotient (AQ): Evidence from Asperger Syndrome/High-Functioning Autism, Malesand Females, Scientists and Mathematicians. *Journal of Autism and Developmental Disorders*, *31*(1), 5–17. https://doi.org/10.1023/A:1005653411471

English, M. C. W., Gignac, G. E., Visser, T. A. W., Whitehouse, A. J. O., Enns, J. T., & Maybery, M. T. (2021). The Comprehensive Autistic Trait Inventory (CATI): Development and validation of a new measure of autistic traits in the general population. *Molecular Autism*, *12*(1), 37. https://doi.org/10.1186/s13229-021-00445-7

Ferris, J., & Wynne, H. (2001). *The Canadian Problem Gambling Index*.

Foa, E. B., Huppert, J. D., Leiberg, S., Langner, R., Kichic, R., Hajcak, G., & Salkovskis, P. M. (2002). The Obsessive-Compulsive Inventory: Development and validation of a short version. *Psychological Assessment*, *14*(4), 485–496. https://doi.org/10.1037/1040-3590.14.4.485

Gillan, C. M., Kosinski, M., Whelan, R., Phelps, E. A., & Daw, N. D. (2016). Characterizing a psychiatric symptom dimension related to deficits in goal-directed control. *eLife*, *5*, e11305. https://doi.org/10.7554/eLife.11305

Heimberg, R. G., Horner, K. J., Juster, H. R., Safren, S. A., Brown, E. J., Schneier, F. R., & Liebowitz, M. R. (1999). Psychometric properties of the Liebowitz Social Anxiety Scale. *Psychological Medicine*, *29*(1), 199–212. https://doi.org/10.1017/S0033291798007879

Lovibond, S. H. (1995). Manual for the depression anxiety stress scales. *Sydney Psychology Foundation*. https://cir.nii.ac.jp/crid/1370294643851494273

Marin, R. S., Biedrzycki, R. C., & Firinciogullari, S. (1991). Reliability and validity of the apathy evaluation scale. *Psychiatry Research*, *38*(2), 143–162. https://doi.org/10.1016/0165-1781(91)90040-V

Mason, O., Linney, Y., & Claridge, G. (2005). Short scales for measuring schizotypy. *Schizophrenia Research*, *78*(2–3), 293–296. https://doi.org/10.1016/j.schres.2005.06.020

Papini, N. M., Jung, M., Cook, A., Lopez, N. V., Ptomey, L. T., Herrmann, S. D., & Kang, M. (2022). Psychometric properties of the 26-item eating attitudes test (EAT-26): An application of rasch analysis. *Journal of Eating Disorders*, *10*(1), 62. https://doi.org/10.1186/s40337-022-00580-3

Patton, J. H., Stanford, M. S., & Barratt, E. S. (1995). Factor structure of the barratt impulsiveness scale. *Journal of Clinical Psychology*, *51*(6), 768–774. https://doi.org/10.1002/1097-4679(199511)51:6%3C768::AID-JCLP2270510607%3E3.0.CO;2-1

Patzelt, E. H., Kool, W., Millner, A. J., & Gershman, S. J. (2019). Incentives Boost Model-Based Control Across a Range of Severity on Several Psychiatric Constructs. *Biological Psychiatry*, *85*(5), 425–433. https://doi.org/10.1016/j.biopsych.2018.06.018

Preece, D., Becerra, R., Robinson, K., Dandy, J., & Allan, A. (2018). *Perth Alexithymia Questionnaire (PAQ): Copy of questionnaire and scoring instructions*.

Revelle, W. (2007). *psych: Procedures for Psychological, Psychometric, and Personality Research* (p. 2.6.1) [Dataset]. https://doi.org/10.32614/CRAN.package.psych

Taylor, S., Zvolensky, M. J., Cox, B. J., Deacon, B., Heimberg, R. G., Ledley, D. R., Abramowitz, J. S., Holaway, R. M., Sandin, B., Stewart, S. H., Coles, M., Eng, W., Daly, E. S., Arrindell, W. A., Bouvard, M., & Cardenas, S. J. (2007). Robust dimensions of anxiety sensitivity: Development and initial validation of the Anxiety Sensitivity Index-3. *Psychological Assessment*, *19*(2), 176–188. https://doi.org/10.1037/1040-3590.19.2.176

Treynor, W. (2003). [No title found]. *Cognitive Therapy and Research*, *27*(3), 247–259. https://doi.org/10.1023/A:1023910315561

Wise, T., Robinson, O. J., & Gillan, C. M. (2023). Identifying Transdiagnostic Mechanisms in Mental Health Using Computational Factor Modeling. *Biological Psychiatry*, *93*(8), 690–703. https://doi.org/10.1016/j.biopsych.2022.09.034

Zung, W. W. K. (1965). A Self-Rating Depression Scale. *Archives of General Psychiatry*, *12*(1), 63. https://doi.org/10.1001/archpsyc.1965.01720310065008

Table A2

Voluntary Task-Switching Analysis With Principal Components

Coefficient	b	Est. Error	l-95% CI	u-95% CI
Intercept	-0.042	0.032	-0.104	0.022
Reward condition	-0.033	0.028	-0.088	0.023
Order	0.031	0.032	-0.032	0.094
D/R	-0.006	0.013	-0.032	0.020
SW	-0.009	0.011	-0.031	0.014
Alexithymia	0.001	0.011	-0.021	0.023
Compulsivity	-0.007	0.010	-0.028	0.013
Age	0.007	0.011	-0.013	0.028
Gender 1	0.014	0.034	-0.052	0.079
Gender 2	0.018	0.034	-0.048	0.085
ICAR	0.008	0.011	-0.013	0.030
Reward condition x Order	-0.018	0.032	-0.083	0.044
Reward condition x D/R	0.002	0.013	-0.024	0.029
Reward condition x SW	-0.004	0.012	-0.027	0.019
Reward condition x Alexithymia	-0.005	0.011	-0.027	0.017
Reward condition x Compulsivity	0.002	0.011	-0.019	0.022
Reward condition x Age	0.002	0.010	-0.018	0.022
Reward condition x Gender 1	0.023	0.030	-0.037	0.082
Reward condition x Gender 2	0.029	0.031	-0.032	0.089
Reward condition x ICAR	0.000	0.011	-0.020	0.021
Order x D/R	0.008	0.014	-0.019	0.034
Order x SW	-0.003	0.012	-0.026	0.019
Order x Alexithymia	-0.007	0.011	-0.028	0.015
Order x Compulsivity	0.002	0.011	-0.019	0.023
Order x Age	-0.006	0.010	-0.026	0.014
Order x Gender 1	-0.036	0.034	-0.102	0.030
Order x Gender 2	-0.025	0.034	-0.092	0.042
Order x ICAR	0.003	0.011	-0.018	0.025
Order x Reward condition x D/R	-0.009	0.014	-0.035	0.018
Order x Reward condition x SW	-0.005	0.012	-0.028	0.018
Order x Reward condition x Alexithymia	-0.005	0.011	-0.026	0.017
Order x Reward condition x Alexithymia	0.000	0.010	-0.021	0.020
Reward condition x Order x Age	-0.001	0.010	-0.022	0.019
Reward condition x Order x Gender 1	0.011	0.033	-0.053	0.077
Reward condition x Order x Gender 2	0.011	0.034	-0.055	0.079
Reward condition x Order x ICAR	0.001	0.011	-0.021	0.021

Note. D/R = Depressive symptoms/Rumination, SW = Social withdrawal

Table A3

Free Choice Accuracy Analyses

Coefficient	b	Est. Error	l-95% CI	u-95% CI
**Intercept**	**3.593**	**0.079**	**3.441**	**3.749**
Reward condition	0.003	0.030	-0.056	0.063
Transition	0.032	0.027	-0.022	0.086
**Interference**	**0.154**	**0.035**	**0.085**	**0.223**
D/R	0.011	0.073	-0.133	0.153
**SW**	**0.143**	**0.067**	**0.013**	**0.275**
**Alexithymia**	**-0.155**	**0.065**	**-0.283**	**-0.028**
**Compulsivity**	**-0.143**	**0.063**	**-0.268**	**-0.020**
Order	-0.016	0.063	-0.139	0.108
Task	0.028	0.056	-0.082	0.138
Reward condition x Transition	-0.032	0.023	-0.078	0.013
Reward condition x Interference	0.004	0.024	-0.043	0.049
Transition x Interference	-0.002	0.024	-0.050	0.045
Reward condition x D/R	-0.040	0.032	-0.101	0.022
Reward condition x SW	0.001	0.030	-0.057	0.059
Reward condition x Alexithymia	0.002	0.029	-0.056	0.059
Reward condition x Compulsivity	0.017	0.028	-0.039	0.072
**Reward condition x Order**	**-0.200**	**0.028**	**-0.255**	**-0.146**
Reward condition x Task	0.025	0.024	-0.021	0.072
Transition x D/R	0.007	0.028	-0.047	0.061
Transition x SW	-0.023	0.026	-0.074	0.027
Transition x Alexithymia	0.025	0.026	-0.027	0.075
Transition x Compulsivity	-0.016	0.025	-0.066	0.033
Transition x Order	0.026	0.025	-0.022	0.075
Transition x Task	-0.007	0.024	-0.055	0.040
Interference x D/R	0.015	0.037	-0.057	0.087
Interference x SW	0.017	0.034	-0.051	0.085
Interference x Alexithymia	0.021	0.034	-0.045	0.088
Interference x Compulsivity	-0.017	0.033	-0.081	0.047
Interference x Order	0.023	0.032	-0.040	0.087
Interference x Task	0.017	0.024	-0.030	0.064
Reward condition x Transition x D/R	-0.028	0.026	-0.079	0.023
Reward condition x Transition x SW	-0.007	0.024	-0.054	0.040
Reward condition x Transition x Alexithymia	0.007	0.024	-0.040	0.055
Reward condition x Transition x Compulsivity	-0.013	0.023	-0.059	0.033
**Reward condition x Transition x Order**	**0.052**	**0.023**	**0.007**	**0.096**
Reward condition x Transition x Task	-0.002	0.022	-0.046	0.041
Reward condition x Interference x D/R	-0.035	0.026	-0.087	0.015
Reward condition x Interference x SW	-0.038	0.024	-0.085	0.009
**Reward condition x Interference x Alexithymia**	**0.055**	**0.024**	**0.007**	**0.102**
Reward condition x Interference x Compulsivity	-0.036	0.023	-0.082	0.009
Reward condition x Interference x Order	0.005	0.023	-0.039	0.049
Reward condition x Interference x Task	-0.034	0.022	-0.077	0.009
Transition x Interference x D/R	0.028	0.026	-0.024	0.078
Transition x Interference x SW	-0.009	0.024	-0.057	0.039
Transition x Interference x Alexithymia	-0.014	0.024	-0.062	0.034
Transition x Interference x Compulsivity	-0.008	0.024	-0.055	0.039
Transition x Interference x Order	0.020	0.023	-0.026	0.065
Transition x Interference x Task	0.005	0.022	-0.039	0.049

Note. D/R = Depressive symptoms/Rumination, SW = Social withdrawal

Table A4

Cued Choice Accuracy

Coefficient	b	Est. Error	l-95% CI	u-95% CI
**Intercept**	**2.705**	**0.144**	**2.425**	**2.990**
Reward condition	-0.082	0.069	-0.217	0.053
**Transition**	**0.142**	**0.061**	**0.024**	**0.263**
Interference	0.141	0.076	-0.007	0.289
D/R	-0.006	0.053	-0.111	0.098
**SW**	**0.111**	**0.048**	**0.017**	**0.205**
Alexithymia	-0.056	0.048	-0.150	0.037
Compulsivity	-0.024	0.045	-0.110	0.064
Order	0.010	0.043	-0.075	0.096
**Task**	**0.105**	**0.042**	**0.024**	**0.186**
Age	0.048	0.045	-0.041	0.136
**ICAR**	**0.246**	**0.046**	**0.157**	**0.336**
Gender 1	0.026	0.145	-0.263	0.310
Gender 2	-0.056	0.146	-0.343	0.227
Reward condition x Transition	0.026	0.058	-0.088	0.140
Reward condition x Interference	-0.010	0.057	-0.122	0.105
Transition x Interference	-0.051	0.058	-0.166	0.063
Reward condition x D/R	0.010	0.025	-0.039	0.060
Reward condition x SW	0.012	0.023	-0.033	0.056
Reward condition x Alexithymia	0.011	0.023	-0.034	0.056
Reward condition x Compulsivity	0.003	0.021	-0.038	0.045
**Reward condition x Order**	**-0.221**	**0.021**	**-0.262**	**-0.180**
Reward condition x Task	-0.010	0.018	-0.044	0.025
Reward condition x Age	-0.024	0.021	-0.065	0.016
Reward condition x ICAR	0.009	0.022	-0.033	0.052
Reward condition x Gender 1	0.107	0.071	-0.033	0.246
Reward condition x Gender 2	0.062	0.072	-0.077	0.203
Transition x D/R	0.018	0.021	-0.024	0.061
Transition x SW	0.023	0.020	-0.015	0.062
Transition x Alexithymia	0.008	0.020	-0.032	0.047
**Transition x Compulsivity**	**0.043**	**0.018**	**0.007**	**0.080**
Transition x Order	0.018	0.018	-0.017	0.054
Transition x Task	0.031	0.018	-0.004	0.065
Transition x Age	-0.013	0.018	-0.048	0.022
Transition x ICAR	0.002	0.019	-0.035	0.039
Transition x Gender 1	-0.074	0.063	-0.199	0.047
Transition x Gender 2	-0.121	0.063	-0.246	0.001
Interference x D/R	0.012	0.028	-0.043	0.065
Interference x SW	-0.008	0.025	-0.058	0.042
Interference x Alexithymia	0.009	0.025	-0.040	0.059
Interference x Compulsivity	-0.025	0.023	-0.071	0.021
Interference x Order	-0.013	0.023	-0.058	0.032
Interference x Task	-0.009	0.018	-0.045	0.026
Interference x Age	0.031	0.023	-0.013	0.076
Interference x ICAR	0.008	0.024	-0.039	0.055
Interference x Gender 1	-0.019	0.078	-0.172	0.133
Interference x Gender 2	-0.017	0.078	-0.169	0.137
Reward condition x Transition x D/R	0.002	0.021	-0.039	0.042
Reward condition x Transition x SW	0.008	0.019	-0.029	0.046
Reward condition x Transition x Alexithymia	0.007	0.019	-0.030	0.045
Reward condition x Transition x Compulsivity	0.020	0.018	-0.014	0.055
Reward condition x Transition x Order	-0.028	0.017	-0.062	0.006
**Reward condition x Transition x Task**	**-0.046**	**0.017**	**-0.080**	**-0.012**
Reward condition x Transition x Age	-0.028	0.017	-0.061	0.006
Reward condition x Transition x ICAR	0.031	0.018	-0.005	0.066
Reward condition x Transition x Gender 1	-0.037	0.060	-0.156	0.081
Reward condition x Transition x Gender 2	-0.043	0.061	-0.161	0.076
Reward condition x Interference x D/R	0.032	0.021	-0.008	0.073
Reward condition x Interference x SW	-0.012	0.019	-0.050	0.025

Coefficient	b	Est. Error	l-95% CI	u-95% CI
Reward condition x Interference x Alexithymia	-0.002	0.020	-0.040	0.036
Reward condition x Interference x Compulsivity	0.002	0.018	-0.034	0.037
Reward condition x Interference x Order	-0.017	0.017	-0.051	0.017
Reward condition x Interference x Task	0.000	0.017	-0.034	0.034
Reward condition x Interference x Age	0.008	0.017	-0.026	0.041
Reward condition x Interference x ICAR	-0.010	0.018	-0.046	0.026
Reward condition x Interference x Gender 1	-0.018	0.059	-0.136	0.098
Reward condition x Interference x Gender 2	-0.008	0.060	-0.126	0.108
Transition x Interference x D/R	-0.021	0.021	-0.062	0.019
Transition x Interference x SW	0.007	0.019	-0.030	0.044
Transition x Interference x Alexithymia	-0.004	0.019	-0.042	0.034
Transition x Interference x Compulsivity	-0.013	0.018	-0.048	0.022
Transition x Interference x Order	-0.007	0.017	-0.041	0.027
Transition x Interference x Task	-0.002	0.017	-0.036	0.032
**Transition x Interference x Age**	**-0.035**	**0.017**	**-0.069**	**-0.001**
Transition x Interference x ICAR	0.013	0.018	-0.023	0.049
Transition x Interference x Gender 1	0.044	0.060	-0.074	0.161
Transition x Interference x Gender 2	-0.008	0.060	-0.127	0.111

Note. D/R = Depressive symptoms/Rumination, SW = Social withdrawal

Table A5

Free Choice RT Analyses

Coefficient	b	Est. Error	l-95% CI	u-95% CI
**Intercept**	**6.711**	**0.057**	**6.600**	**6.822**
Reward condition	0.002	0.013	-0.024	0.028
**Transition**	**-0.017**	**0.008**	**-0.032**	**-0.002**
**Interference**	**-0.015**	**0.005**	**-0.025**	**-0.004**
D/R	-0.016	0.023	-0.060	0.028
SW	0.005	0.020	-0.034	0.044
Alexithymia	-0.013	0.019	-0.051	0.025
Compulsivity	0.000	0.018	-0.036	0.036
Order	-0.017	0.018	-0.053	0.018
**Task**	**-0.022**	**0.004**	**-0.029**	**-0.015**
Age	0.017	0.018	-0.019	0.053
**ICAR**	**-0.047**	**0.019**	**-0.084**	**-0.010**
Gender 1	-0.039	0.058	-0.153	0.075
Gender 2	0.055	0.059	-0.060	0.170
Reward condition x Transition	-0.002	0.005	-0.011	0.008
Reward condition x Interference	-0.005	0.005	-0.013	0.004
Transition x Interference	0.005	0.005	-0.004	0.014
Reward condition x D/R	0.002	0.005	-0.008	0.013
Reward condition x SW	0.002	0.005	-0.007	0.012
Reward condition x Alexithymia	-0.008	0.005	-0.017	0.002
Reward condition x Compulsivity	-0.002	0.004	-0.010	0.007
**Reward condition x Order**	**0.041**	**0.004**	**0.032**	**0.049**
Reward condition x Task	0.001	0.002	-0.002	0.004
Reward condition x Age	-0.004	0.004	-0.013	0.004
Reward condition x ICAR	0.000	0.004	-0.009	0.009
Reward condition x Gender 1	-0.011	0.014	-0.038	0.016
Reward condition x Gender 2	-0.007	0.014	-0.033	0.020
Transition x D/R	-0.001	0.003	-0.007	0.006
Transition x SW	0.005	0.003	0.000	0.011
**Transition x Alexithymia**	**-0.007**	**0.003**	**-0.012**	**-0.001**
Transition x Compulsivity	-0.003	0.003	-0.008	0.002
Transition x Order	0.003	0.002	-0.002	0.008
Transition x Task	0.002	0.002	-0.001	0.005
Transition x Age	-0.005	0.002	-0.009	0.000
**Transition x ICAR**	**0.007**	**0.003**	**0.002**	**0.012**
Transition x Gender 1	-0.011	0.008	-0.026	0.004
Transition x Gender 2	-0.011	0.008	-0.027	0.004
Interference x D/R	-0.004	0.002	-0.008	0.000
Interference x SW	0.002	0.002	-0.002	0.006
Interference x Alexithymia	-0.001	0.002	-0.005	0.003
Interference x Compulsivity	0.001	0.002	-0.003	0.004
Interference x Order	0.000	0.002	-0.003	0.004
Interference x Task	0.001	0.002	-0.002	0.004
Interference x Age	-0.001	0.002	-0.005	0.002
Interference x ICAR	-0.002	0.002	-0.005	0.002
Interference x Gender 1	0.007	0.006	-0.004	0.018
Interference x Gender 2	0.005	0.006	-0.006	0.016
Reward condition x Transition x D/R	-0.002	0.002	-0.006	0.002
Reward condition x Transition x SW	0.000	0.002	-0.004	0.003
Reward condition x Transition x Alexithymia	-0.001	0.002	-0.005	0.002
Reward condition x Transition x Compulsivity	0.000	0.002	-0.003	0.003
Reward condition x Transition x Order	-0.002	0.002	-0.006	0.001
Reward condition x Transition x Task	-0.001	0.001	-0.004	0.002
Reward condition x Transition x Age	0.002	0.002	-0.001	0.005
Reward condition x Transition x ICAR	0.002	0.002	-0.002	0.005
Reward condition x Transition x Gender 1	0.003	0.005	-0.007	0.013
Reward condition x Transition x Gender 2	-0.001	0.005	-0.011	0.009
**Reward condition x Interference x D/R**	**-0.005**	**0.002**	**-0.009**	**-0.001**
Reward condition x Interference x S/W	0.002	0.002	-0.001	0.005

Coefficient	b	Est. Error	l-95% CI	u-95% CI
Reward condition x Interference x Alexithymia	0.003	0.002	-0.001	0.006
Reward condition x Interference x Compulsivity	0.000	0.002	-0.003	0.003
Reward condition x Interference x Order	0.002	0.002	-0.001	0.005
Reward condition x Interference x Task	0.002	0.001	-0.001	0.005
Reward condition x Interference x Age	-0.001	0.002	-0.004	0.002
Reward condition x Interference x ICAR	-0.001	0.002	-0.004	0.002
Reward condition x Interference x Gender 1	0.006	0.005	-0.004	0.015
Reward condition x Interference x Gender 2	0.005	0.005	-0.004	0.014
Transition x Interference x D/R	-0.002	0.002	-0.005	0.002
Transition x Interference x SW	0.000	0.002	-0.004	0.003
Transition x Interference x Alexithymia	-0.001	0.002	-0.004	0.002
**Transition x Interference x Compulsivity**	**0.004**	**0.002**	**0.001**	**0.007**
Transition x Interference x Order	0.002	0.002	-0.001	0.005
Transition x Interference x Task	-0.001	0.002	-0.004	0.002
Transition x Interference x Age	0.000	0.002	-0.003	0.003
Transition x Interference x ICAR	0.000	0.002	-0.004	0.003
Transition x Interference x Gender 1	-0.004	0.005	-0.014	0.005
Transition x Interference x Gender 2	-0.005	0.005	-0.014	0.005

Note. D/R = Depressive symptoms/Rumination, SW = Social withdrawal

Table A6

Cued Choice RT

Coefficient	b	Est. Error	l-95% CI	u-95% CI
**Intercept**	**6.264**	**0.067**	**6.131**	**6.396**
Reward condition	-0.002	0.016	-0.034	0.030
**Transition**	**-0.028**	**0.010**	**-0.048**	**-0.009**
Interference	0.001	0.008	-0.015	0.017
D/R	-0.023	0.092	-0.204	0.159
SW	0.109	0.065	-0.018	0.236
Alexithymia	-0.084	0.063	-0.207	0.038
**Compulsivity**	**0.155**	**0.071**	**0.017**	**0.293**
Order	-0.033	0.020	-0.073	0.006
**Task**	**-0.042**	**0.005**	**-0.052**	**-0.032**
**Age**	**0.059**	**0.020**	**0.019**	**0.099**
**ICAR**	**-0.063**	**0.020**	**-0.103**	**-0.023**
Gender 1	-0.034	0.065	-0.160	0.094
**Gender 2**	**0.132**	**0.064**	**0.006**	**0.258**
Reward condition x Transition	0.002	0.007	-0.012	0.015
Reward condition x Interference	0.002	0.007	-0.011	0.016
Transition x Interference	0.006	0.007	-0.008	0.019
Reward condition x D/R	-0.003	0.023	-0.047	0.042
Reward condition x SW	-0.002	0.016	-0.033	0.029
Reward condition x Alexithymia	0.011	0.015	-0.019	0.040
Reward condition x Compulsivity	-0.012	0.017	-0.045	0.022
**Reward condition x Order**	**0.051**	**0.005**	**0.042**	**0.061**
Reward condition x Task	0.001	0.002	-0.003	0.005
Reward condition x Age	-0.006	0.005	-0.015	0.004
Reward condition x ICAR	-0.001	0.005	-0.011	0.009
Reward condition x Gender 1	-0.004	0.016	-0.035	0.027
Reward condition x Gender 2	0.003	0.016	-0.028	0.034
Transition x D/R	-0.020	0.014	-0.047	0.007
Transition x SW	0.001	0.010	-0.018	0.020
Transition x Alexithymia	-0.003	0.009	-0.020	0.015
Transition x Compulsivity	-0.013	0.010	-0.033	0.008
Transition x Order	-0.001	0.003	-0.007	0.005
**Transition x Task**	**-0.009**	**0.002**	**-0.013**	**-0.005**
Transition x Age	-0.001	0.003	-0.007	0.004
Transition x ICAR	-0.002	0.003	-0.008	0.004
Transition x Gender 1	-0.002	0.010	-0.021	0.016
Transition x Gender 2	-0.004	0.009	-0.022	0.015
Interference x D/R	-0.001	0.011	-0.023	0.020
Interference x SW	-0.002	0.008	-0.017	0.013
Interference x Alexithymia	0.002	0.007	-0.012	0.017
Interference x Compulsivity	-0.002	0.008	-0.018	0.014
Interference x Order	-0.001	0.002	-0.006	0.003
Interference x Task	0.000	0.002	-0.004	0.005
Interference x Age	0.001	0.002	-0.003	0.006
Interference x ICAR	0.002	0.002	-0.003	0.006
Interference x Gender 1	-0.009	0.008	-0.024	0.006
Interference x Gender 2	0.000	0.008	-0.015	0.015
Reward condition x Transition x D/R	0.002	0.010	-0.017	0.020
Reward condition x Transition x SW	0.000	0.007	-0.013	0.014
Reward condition x Transition x Alexithymia	0.005	0.006	-0.007	0.018
Reward condition x Transition x Compulsivity	0.000	0.007	-0.015	0.014
**Reward condition x Transition x Order**	**-0.006**	**0.002**	**-0.010**	**-0.002**
Reward condition x Transition x Task	-0.003	0.002	-0.007	0.001
Reward condition x Transition x Age	0.000	0.002	-0.004	0.005
Reward condition x Transition x ICAR	0.002	0.002	-0.002	0.006
Reward condition x Transition x Gender 1	-0.002	0.007	-0.015	0.011
Reward condition x Transition x Gender 2	-0.007	0.007	-0.020	0.006
Reward condition x Interference x D/R	0.000	0.010	-0.019	0.019
Reward condition x Interference x S/W	-0.010	0.007	-0.023	0.003

Coefficient	b	Est. Error	l-95% CI	u-95% CI
Reward condition x Interference x Alexithymia	-0.001	0.006	-0.014	0.011
Reward condition x Interference x Compulsivity	-0.008	0.007	-0.022	0.007
Reward condition x Interference x Order	0.003	0.002	-0.001	0.007
Reward condition x Interference x Task	-0.001	0.002	-0.005	0.004
Reward condition x Interference x Age	0.000	0.002	-0.004	0.004
Reward condition x Interference x ICAR	0.001	0.002	-0.003	0.005
Reward condition x Interference x Gender 1	0.003	0.007	-0.010	0.016
Reward condition x Interference x Gender 2	0.000	0.007	-0.013	0.014
Transition x Interference x D/R	-0.006	0.010	-0.025	0.013
Transition x Interference x SW	0.003	0.007	-0.010	0.016
Transition x Interference x Alexithymia	0.002	0.006	-0.011	0.014
Transition x Interference x Compulsivity	-0.008	0.007	-0.022	0.007
Transition x Interference x Order	-0.001	0.002	-0.005	0.003
Transition x Interference x Task	0.001	0.002	-0.003	0.005
Transition x Interference x Age	0.000	0.002	-0.004	0.004
Transition x Interference x ICAR	0.000	0.002	-0.004	0.004
Transition x Interference x Gender 1	-0.005	0.007	-0.018	0.008
Transition x Interference x Gender 2	0.001	0.007	-0.012	0.014

Note. D/R = Depressive symptoms/Rumination, SW = Social withdrawal

Appendix B

Table B1

The Educational Level of the Sample Including Pilot Participants (N=418)

**Level obtained**	**N** $_{total}$	**N** $_{autism}$	**N** $_{control}$
No formal qualifications	2	0	2
Secondary education (e.g. GED/GCSE)	29	7	22
High school diploma/A-levels	112	28	84
Technical/community college	40	3	37
Undergraduate degree (BA/BSc/other)	161	21	140
Graduate degree (MA/MSc/MPhil/other)	69	8	61
Doctorate degree (PhD/other)	5	5	0

Table B2

The Employment Status of the Sample Including Pilot Participants (N=418)

**Employment**	**N** $_{total}$	**N** $_{autism}$	**N** $_{control}$
Disabled (not working because of permanent or temporary disability)	17	9	8
Homemaker	18	1	17
Unemployed, looking for work	72	18	54
Due to start a new job within the next month	11	3	8
Working parttime	83	17	66
Working fulltime	217	19	198

Table B3

Annual Income of the Sample Including Pilot Participants (N=418)

Income	Currency	N total	N autism	N control
Less than 10,000	GPB	23	6	17
	USD	14	8	6
10,000 - 15,999	GPB	18	4	14
	USD	8	1	7
16,000 - 19,999	GPB	22	2	20
	USD	1	0	1
20,000 - 29,999	GPB	46	11	35
	USD	8	0	8
30,000 - 39,999	GPB	49	8	41
	USD	14	4	10
40,000 - 49,999	GPB	46	4	42
	USD	9	2	7
50,000 - 59,999	GPB	35	5	30
	USD	13	3	10
60,000 - 69,999	GPB	18	1	17
	USD	7	4	3
70,000 - 79,999	GPB	16	0	16
	USD	4	0	4
80,000 - 89,999	GPB	11	0	11
	USD	7	0	7
90,000 - 99,999	GPB	9	0	9
	USD	13	1	12
100,000 - 149,999	GPB	5	0	5
	USD	16	2	14
More than 150,000	GPB	2	0	2
	USD	4	1	3

Appendix C

Task instructions

In this task, you will perform two different categorization tasks indicated by different letter cues. At the start of each trial, a fixation cross will appear in the centre of the screen, followed by a letter cue. If this letter cue is a vowel (= letter A, E, I, O or U), you will need to categorize a following word on its <task 1>. If the letter cue is a consonant (= letter V, F, L, Q or C), you will need to categorize the word on its <task 2>.

In the size task, we ask you to judge whether the word presented is smaller or larger than a basketball. If it is smaller than a basketball, press the '<smaller button>' button, if it is larger than a basketball, press the '<larger button>' button.

In the animacy task, we ask you to judge whether the word is animate or inanimate. If the word is <animacy condition 1>, press the '<animacy condition 1 button>' button. If the word is <animacy condition 2>, press the '<animacy condition 2 button' button.

Please note that animate refers to any kind of organism: animal, tree, plant, nut, fruit or vegetable. This means you will use your left hand for the <task1> task and your right hand for the <task2> task. You can win additional money when you are responding accurately and fast enough, that we will pay you as a bonus payment after finishing all three sessions. Specifically, for each trial, if you respond correctly, you will gain a randomly determined reward of either 1 or 10 points. Every 3 points are worth 1 penny, which means you can receive up to an additional 2.9 pounds! (Or the equivalent converted to $ if you're paid in dollars.) This will be paid out if you complete all three sessions.

Sometimes, the letter cue is replaced by the # symbol. This means that you can randomly choose which task to do on that trial. Your choice for which task to do should be random as if you flipped a coin. On these trials, you cannot win points, but accurate performance and an honest attempt to choose tasks randomly is important. In other words, a failure to respond accurately on these trials, or a failure to choose tasks randomly (for example, constantly choosing the same task), would mean that you might not receive your payment.

To remind you, if the letter cue is a vowel, you will need to do a <task 1> task: the '<task 1 condition 1 button>' button means <task 1 condition 1>, and the 'task 1 condition 2' button means <task 1 condition 2>.

If the letter cue is a consonant, you will need to do a <task 2> task: the '<task 2 condition 1 button>' button means <task 2 condition 1>, and the '<task 2 condition 2 button>' button means <task 2 condition 2>.

You can start with a practice phase now, which doesn't include rewards yet. If your accuracy in the practice phase is high enough, you will proceed to the actual experiment afterwards. Otherwise, you will need to repeat the practice phase. Click 'next' to immediately start with the practice phase.

[Conditional practice phase with repetition if accuracy below criterion]

This is the end of the practice phase. Your accuracy in the practice phase was <accuracy> %. You can take a break now, and then continue with the actual experiment. Another reminder: when the letter cue is replaced by the # symbol, it means that you can randomly choose which task to do on that trial. Click 'next' to start with the actual experiment now.

[…]

Appendix D

Table 1

Split-Half Reliability of Free Choice and Performance Indicators on Free and Cued Choice Trials

Effect	Measure	Phase	Reliability coefficient
**Voluntary switch rate**	Task choice	Free	0.884
**Switch cost**	RT	Free	0.586
		Cued	0.354
	Accuracy	Free	0.111
		Cued	0.154
**Interference effect**	RT	Free	0.212
		Cued	0.239
	Accuracy	Free	0.463
		Cued	0.350

Note. Reliabilities were obtained by dividing Pearson correlations between the costs of odd/even trials by the mean accuracy/RT per subject and applying the Spearman-Brown prophecy formula (Brown, 1910; Spearman, 1910).

Appendix E

Table E1

Raw voluntary switch rate per variable level in the voluntary task-switching group model

Variable	level	Mean	SD
Reward condition	Repeat reinforced	0.503	0.172
	Switch reinforced	0.500	0.174
Order	Repeat first	0.491	0.145
	Switch first	0.510	0.174
Group	Autism	0.542	0.163
	Control	0.492	0.159
Age	Low (lowest tertile)	0.520	0.157
	Medium	0.500	0.155
	High	0.480	0.168
Gender	Women	0.486	0.152
	Men	0.509	0.164
	Other	0.582	0.209
ICAR	Low (lowest tertile)	0.452	0.155
	Medium	0.497	0.140
	High	0.552	0.170

Table E2

Raw accuracy per variable level in the cued choice group model

Variable	level	Mean	SD
Reward condition	Repeat reinforced	0.890	0.080
	Switch reinforced	0.895	0.075
Transition	Repetition	0.898	0.069
	Switch	0.890	0.076
Interference	Non-interfering	0.902	0.072
	interfering	0.886	0.080
Group	Autism	0.899	0.064
	Control	0.893	0.068
Order	Repeat first	0.894	0.066
	Switch first	0.893	0.070
Task	Animacy	0.897	0.078
	Size	0.891	0.076
Age	Low (lowest tertile)	0.887	0.071
	Medium	0.899	0.064
	High	0.896	0.067
ICAR	Low (lowest tertile)	0.871	0.068
	Medium	0.892	0.067
	High	0.919	0.060
Gender	Women	0.895	0.068
	Men	0.892	0.069
	Other	0.902	0.042

Table E3

Raw accuracy per variable level in the free choice group model

Variable	level	Mean	SD
Reward condition	Repeat reinforced	0.935	0.076
	Switch reinforced	0.934	0.078
Transition	Repetition	0.940	0.071
	Switch	0.932	0.075
Interference	Non-interfering	0.942	0.076
	Interfering	0.929	0.077
Group	Autism	0.938	0.072
	Control	0.935	0.071

Order	Repeat first	0.933	0.076
	Switch first	0.938	0.065
Task	Animacy	0.933	0.077
	Size	0.937	0.076
Age	Low (lowest tertile)	0.928	0.069
	Medium	0.937	0.083
	High	0.941	0.057
ICAR	Low (lowest tertile)	0.922	0.080
	Medium	0.938	0.055
	High	0.946	0.073
Gender	Women	0.939	0.058
	Men	0.931	0.083
	Other	0.943	0.033

Table E4

Raw Reaction times in the cued choice group model

**Variable**	**level**	**Mean**	**SD**
Reward condition	Repeat reinforced	1002	304
	Switch reinforced	1013	324
Transition	Repetition	976	290
	Switch	1034	323
Interference	Non-interfering	1005	308
	Interfering	1006	305
Group	Autism	1095	325
	Control	988	298
Order	Repeat first	977	300
	Switch first	1034	307
Task	Animacy	989	310
	Size	1025	311
Age	Low (lowest tertile)	999	331
	Medium	998	306
	High	1019	276
ICAR	Low (lowest tertile)	1049	336
	Medium	1021	289
	High	946	278
Gender	Women	956	286
	Men	1049	313
	Other	1098	340

Table E5

Raw reaction times in the free choice group model

**Variable**	**level**	**Mean**	**SD**
Reward condition	Repeat reinforced	1081	307
	Switch reinforced	1089	305
Transition	Repetition	1065	294
	Switch	1122	312
Interference	Non-interfering	1080	301
	Interfering	1090	291
Group	Autism	1180	304
	Control	1066	289
Order	Repeat first	1061	299
	Switch first	1108	288
Task	Animacy	1074	304
	Size	1106	299
Age	Low (lowest tertile)	1092	335
	Medium	1073	289
	High	1089	254
ICAR	Low (lowest tertile)	1091	294

	Medium	1127	279
	High	1036	303
Gender	Women	1028	269
	Men	1137	310
	Other	1139	250

Appendix F

Table F1

Group Analysis Voluntary Task Switching

Coefficient	b	Est. Error	l-95% CI	u-95% CI
**Intercept**	**0.184**	**0.082**	**0.021**	**0.347**
Reward condition	-0.007	0.028	-0.062	0.046
**Order**	**-0.230**	**0.082**	**-0.388**	**-0.069**
Group	0.085	0.058	-0.028	0.197
Age	-0.033	0.040	-0.112	0.045
Gender 1	-0.118	0.090	-0.297	0.055
Gender 2	-0.075	0.088	-0.249	0.097
**ICAR**	**0.203**	**0.040**	**0.124**	**0.281**
Reward condition x Order	0.017	0.032	-0.046	0.080
Reward condition x Group	-0.011	0.022	-0.053	0.031
Reward condition x Age	0.026	0.015	-0.004	0.056
Reward condition x Gender 1	0.012	0.033	-0.053	0.077
Reward condition x Gender 2	0.007	0.032	-0.056	0.070
Reward condition x ICAR	0.020	0.015	-0.010	0.050
Order x Group	-0.030	0.057	-0.142	0.082
Order x Age	-0.021	0.041	-0.100	0.059
Order x Gender 1	0.162	0.092	-0.024	0.342
Order x Gender 2	0.157	0.090	-0.021	0.329
Order x ICAR	-0.018	0.040	-0.095	0.061
Reward condition x Order x Group	0.005	0.021	-0.038	0.047
Reward condition x Order x Age	0.017	0.015	-0.013	0.047
Reward condition x Order x Gender 1	0.022	0.035	-0.047	0.091
Reward condition x Order x Gender 2	-0.001	0.034	-0.068	0.066
Reward condition x Order x ICAR	-0.006	0.015	-0.036	0.023

Table F2

Group Analysis Voluntary Task-Switching First Experiment Half

Coefficient	b	Est. Error	l-95% CI	u-95% CI
**Intercept**	**0.174**	**0.075**	**0.028**	**0.323**
**Order**	**-0.192**	**0.076**	**-0.343**	**-0.041**
Group	0.087	0.052	-0.013	0.189
Age	-0.081	0.082	-0.246	0.080
Gender 1	-0.074	0.081	-0.234	0.082
Gender 2	-0.020	0.037	-0.094	0.052
**ICAR**	**0.190**	**0.037**	**0.120**	**0.263**
Order x Group	-0.038	0.053	-0.142	0.066
Order x Age	0.143	0.082	-0.020	0.304
Order x Gender 1	0.133	0.082	-0.027	0.295
Order x Gender 2	0.008	0.038	-0.067	0.082
Order x ICAR	0.001	0.037	-0.071	0.074

Table F3

Trait Analysis Voluntary Task-Switching

Coefficient	b	Est. Error	l-95% CI	u-95% CI
Intercept	0.108	0.140	-0.169	0.381
Reward condition	-0.041	0.039	-0.118	0.035
Order	-0.050	0.137	-0.314	0.216
Traits	-0.018	0.039	-0.093	0.058
Age	-0.018	0.043	-0.103	0.064
Gender 1	-0.130	0.146	-0.414	0.154
Gender 2	-0.071	0.145	-0.349	0.216
**ICAR**	**0.210**	**0.044**	**0.125**	**0.295**
Reward condition x Order	-0.036	0.050	-0.134	0.063
Reward condition x Traits	-0.004	0.014	-0.032	0.024
Reward condition x Age	0.028	0.016	-0.003	0.059
Reward condition x Gender 1	0.064	0.043	-0.019	0.148
Reward condition x Gender 2	0.046	0.043	-0.038	0.129
Reward condition x ICAR	0.022	0.016	-0.010	0.054
Order x Traits	0.051	0.039	-0.025	0.128
Order x Age	-0.047	0.042	-0.130	0.037
Order x Gender 1	-0.045	0.141	-0.321	0.228
Order x Gender 2	-0.017	0.143	-0.295	0.260
Order x ICAR	-0.057	0.043	-0.142	0.027
Reward condition x Order x Traits	0.001	0.014	-0.027	0.029
Reward condition x Order x Age	0.017	0.016	-0.014	0.048
Reward condition x Order x Gender 1	0.073	0.052	-0.028	0.175
Reward condition x Order x Gender 2	0.040	0.052	-0.063	0.141
Reward condition x Order x ICAR	0.005	0.016	-0.026	0.037

Appendix G

Table G1

Group Analysis Free Choice Accuracy

Coefficient	b	Est. Error	l-95% CI	u-95% CI
**Intercept**	**3.622**	**0.122**	**3.385**	**3.863**
Reward condition	0.011	0.051	-0.090	0.112
Transition	0.010	0.046	-0.081	0.100
**Interference**	**0.173**	**0.058**	**0.059**	**0.288**
Group	0.097	0.080	-0.059	0.255
Order	-0.054	0.054	-0.161	0.052
Task	0.012	0.051	-0.088	0.112
**Age**	**0.163**	**0.056**	**0.052**	**0.274**
**ICAR**	**0.325**	**0.057**	**0.214**	**0.436**
Gender 1	0.126	0.125	-0.121	0.372
Gender 2	-0.080	0.122	-0.320	0.159
Reward condition x Transition	-0.019	0.042	-0.101	0.063
Reward condition x Interference	0.039	0.043	-0.045	0.123
Transition x Interference	-0.040	0.043	-0.126	0.045
Reward condition x Group	0.056	0.036	-0.014	0.126
**Reward condition x Order**	**-0.195**	**0.024**	**-0.242**	**-0.148**
Reward condition x Task	0.006	0.021	-0.034	0.046
**Reward condition x Age**	**-0.056**	**0.024**	**-0.103**	**-0.009**
Reward condition x ICAR	-0.020	0.025	-0.069	0.029
Reward condition x Gender 1	0.057	0.056	-0.052	0.165
Reward condition x Gender 2	0.062	0.054	-0.043	0.169
Transition x Group	0.041	0.031	-0.019	0.102
Transition x Order	0.023	0.021	-0.018	0.064
Transition x Task	-0.003	0.021	-0.044	0.037
Transition x Age	0.004	0.021	-0.037	0.044
Transition x ICAR	0.002	0.022	-0.041	0.045
Transition x Gender 1	0.055	0.050	-0.044	0.153
Transition x Gender 1	0.073	0.049	-0.022	0.170
Interference x Group	-0.007	0.041	-0.087	0.074
Interference x Order	0.019	0.028	-0.036	0.074
Interference x Task	0.009	0.021	-0.033	0.051
Interference x Age	-0.013	0.028	-0.068	0.042
Interference x ICAR	0.027	0.029	-0.029	0.085
Interference x Gender 1	-0.011	0.063	-0.135	0.112
Interference x Gender 2	-0.039	0.061	-0.160	0.081
Reward condition x Transition x Group	0.039	0.030	-0.019	0.098
Reward condition x Transition x Order	0.035	0.020	-0.004	0.074
Reward condition x Transition x Task	0.006	0.020	-0.032	0.044
Reward condition x Transition x Age	-0.004	0.020	-0.043	0.034
**Reward condition x Transition x ICAR**	**-0.044**	**0.021**	**-0.085**	**-0.003**
Reward condition x Transition x Gender 1	0.017	0.046	-0.074	0.107
Reward condition x Transition x Gender 2	0.021	0.045	-0.067	0.108
Reward condition x Interference x Group	0.025	0.030	-0.033	0.083
Reward condition x Interference x Order	-0.011	0.020	-0.049	0.028
Reward condition x Interference x Task	-0.034	0.019	-0.072	0.004
Reward condition x Interference x Age	0.001	0.020	-0.037	0.039
Reward condition x Interference x ICAR	0.006	0.020	-0.033	0.046
Reward condition x Interference x Gender 1	-0.036	0.046	-0.127	0.054
Reward condition x Interference x Gender 2	-0.028	0.045	-0.117	0.060
Transition x Interference x Group	-0.002	0.030	-0.062	0.056
Transition x Interference x Order	0.013	0.020	-0.027	0.052
Transition x Interference x Task	0.013	0.019	-0.025	0.051
Transition x Interference x Age	-0.003	0.020	-0.042	0.037
Transition x Interference x ICAR	0.022	0.021	-0.019	0.063
Transition x Interference x Gender 1	0.033	0.047	-0.060	0.126
Transition x Interference x Gender 2	0.038	0.046	-0.052	0.128

Table G2

Trait Analysis Free Choice Accuracy

Coefficient	b	Est. Error	l-95% CI	u-95% CI
**Intercept**	**3.574**	**0.078**	**3.421**	**3.729**
Reward condition	0.008	0.029	-0.050	0.065
Transition	0.033	0.026	-0.019	0.083
**Interference**	**0.166**	**0.034**	**0.100**	**0.234**
Traits	0.020	0.055	-0.087	0.129
Order	-0.040	0.061	-0.159	0.080
Task	0.012	0.054	-0.093	0.118
Reward condition x Transition	-0.032	0.022	-0.076	0.012
Reward condition x Interference	-0.022	0.023	-0.066	0.023
Transition x Interference	-0.008	0.023	-0.054	0.037
Reward condition x Traits	0.003	0.023	-0.042	0.049
**Reward condition x Order**	**-0.205**	**0.026**	**-0.256**	**-0.155**
Reward condition x Task	0.022	0.022	-0.022	0.065
Transition x Traits	0.005	0.020	-0.034	0.045
Transition x Order	0.035	0.023	-0.008	0.080
Transition x Task	-0.016	0.023	-0.061	0.028
Interference x Traits	-0.019	0.027	-0.072	0.034
Interference x Order	0.022	0.031	-0.038	0.082
Interference x Task	0.023	0.023	-0.022	0.068
Reward condition x Transition x Traits	0.008	0.019	-0.030	0.045
**Reward condition x Transition x Order**	**0.062**	**0.021**	**0.020**	**0.104**
Reward condition x Transition x Task	0.011	0.021	-0.031	0.052
Reward condition x Interference x Traits	0.030	0.019	-0.007	0.067
Reward condition x Interference x Order	0.005	0.021	-0.036	0.047
Reward condition x Interference x Task	-0.031	0.021	-0.072	0.010
Transition x Interference x Traits	-0.004	0.020	-0.042	0.034
Transition x Interference x Order	0.013	0.022	-0.029	0.055
Transition x Interference x Task	0.012	0.021	-0.030	0.053

Table G3

Group Analysis Cued Choice Accuracy

Coefficient	b	Est. Error	l-95% CI	u-95% CI
**Intercept**	**2.676**	**0.088**	**2.503**	**2.849**
Reward condition	-0.043	0.038	-0.118	0.032
Transition	0.031	0.033	-0.033	0.095
**Interference**	**0.091**	**0.040**	**0.014**	**0.170**
Group	0.062	0.058	-0.051	0.177
Order	0.001	0.039	-0.076	0.079
**Task**	**0.078**	**0.038**	**0.004**	**0.154**
**Age**	**0.082**	**0.040**	**0.003**	**0.160**
**ICAR**	**0.293**	**0.041**	**0.213**	**0.374**
Gender 1	0.096	0.090	-0.080	0.272
Gender 2	-0.064	0.088	-0.238	0.107
Reward condition x Transition	0.014	0.032	-0.048	0.077
Reward condition x Interference	-0.013	0.032	-0.077	0.050
Transition x Interference	-0.002	0.032	-0.064	0.060
**Reward condition x Group**	**-0.055**	**0.027**	**-0.107**	**-0.003**
**Reward condition x Order**	**-0.204**	**0.018**	**-0.239**	**-0.169**
Reward condition x Task	-0.008	0.016	-0.038	0.023
Reward condition x Age	-0.003	0.019	-0.040	0.033
Reward condition x ICAR	0.006	0.019	-0.031	0.044
Reward condition x Gender 1	-0.009	0.042	-0.089	0.073
Reward condition x Gender 2	-0.033	0.041	-0.113	0.047
Transition x Group	-0.008	0.023	-0.053	0.036
Transition x Order	0.020	0.016	-0.011	0.050
Transition x Task	0.030	0.015	0.000	0.060
Transition x Age	-0.021	0.016	-0.052	0.009
Transition x ICAR	0.003	0.016	-0.028	0.034
Transition x Gender 1	0.025	0.036	-0.045	0.095
Transition x Gender 1	-0.034	0.035	-0.102	0.034
**Interference x Group**	**-0.068**	**0.028**	**-0.123**	**-0.013**
Interference x Order	-0.015	0.019	-0.051	0.022
Interference x Task	-0.002	0.016	-0.033	0.029
Interference x Age	0.002	0.019	-0.035	0.039
Interference x ICAR	-0.012	0.019	-0.050	0.026
Interference x Gender 1	-0.035	0.043	-0.120	0.050
Interference x Gender 2	-0.019	0.042	-0.102	0.064
Reward condition x Transition x Group	0.009	0.022	-0.035	0.052
Reward condition x Transition x Order	-0.023	0.015	-0.052	0.006
**Reward condition x Transition x Task**	**-0.034**	**0.015**	**-0.063**	**-0.004**
Reward condition x Transition x Age	-0.011	0.015	-0.042	0.018
**Reward condition x Transition x ICAR**	**0.038**	**0.016**	**0.007**	**0.069**
Reward condition x Transition x Gender 1	-0.006	0.035	-0.075	0.061
Reward condition x Transition x Gender 2	-0.027	0.034	-0.094	0.040
Reward condition x Interference x Group	-0.011	0.023	-0.055	0.033
Reward condition x Interference x Order	-0.019	0.015	-0.048	0.011
Reward condition x Interference x Task	-0.001	0.015	-0.031	0.028
Reward condition x Interference x Age	-0.013	0.015	-0.043	0.018
Reward condition x Interference x ICAR	-0.025	0.016	-0.056	0.006
Reward condition x Interference x Gender 1	-0.013	0.035	-0.082	0.056
Reward condition x Interference x Gender 2	-0.006	0.034	-0.074	0.062
**Transition x Interference x Group**	**0.047**	**0.022**	**0.003**	**0.091**
Transition x Interference x Order	0.000	0.015	-0.030	0.029
Transition x Interference x Task	0.006	0.015	-0.023	0.035
Transition x Interference x Age	-0.027	0.015	-0.057	0.003
Transition x Interference x ICAR	0.022	0.016	-0.009	0.053
Transition x Interference x Gender 1	0.052	0.035	-0.016	0.120
Transition x Interference x Gender 2	0.013	0.034	-0.054	0.080

Table G4

Trait Analysis Cued Choice Accuracy

Coefficient	b	Est. Error	l-95% CI	u-95% CI
**Intercept**	**2.696**	**0.142**	**2.422**	**2.975**
Reward condition	-0.072	0.067	-0.203	0.060
**Transition**	**0.160**	**0.061**	**0.043**	**0.280**
Interference	0.134	0.073	-0.009	0.279
Traits	-0.034	0.038	-0.107	0.040
Order	-0.007	0.041	-0.087	0.074
**Task**	**0.099**	**0.040**	**0.021**	**0.177**
Age	0.066	0.042	-0.016	0.147
**ICAR**	**0.274**	**0.042**	**0.191**	**0.357**
Gender 1	0.025	0.143	-0.256	0.300
Gender 2	-0.071	0.142	-0.352	0.206
Reward condition x Transition	0.041	0.058	-0.073	0.154
Reward condition x Interference	-0.008	0.057	-0.120	0.105
Transition x Interference	-0.053	0.058	-0.168	0.062
Reward condition x Traits	-0.024	0.018	-0.059	0.011
**Reward condition x Order**	**-0.215**	**0.019**	**-0.252**	**-0.177**
Reward condition x Task	-0.008	0.017	-0.041	0.026
Reward condition x Age	-0.018	0.019	-0.056	0.019
Reward condition x ICAR	0.016	0.020	-0.024	0.056
Reward condition x Gender 1	0.105	0.069	-0.031	0.239
Reward condition x Gender 2	0.064	0.069	-0.071	0.199
**Transition x Traits**	**-0.032**	**0.015**	**-0.062**	**-0.002**
Transition x Order	0.021	0.017	-0.012	0.054
**Transition x Task**	**0.033**	**0.017**	**0.000**	**0.066**
Transition x Age	-0.008	0.016	-0.041	0.024
Transition x ICAR	-0.004	0.018	-0.038	0.031
Transition x Gender 1	-0.087	0.062	-0.210	0.032
**Transition x Gender 2**	**-0.142**	**0.062**	**-0.265**	**-0.022**
Interference x Traits	0.027	0.020	-0.011	0.066
Interference x Order	-0.015	0.022	-0.057	0.028
Interference x Task	-0.003	0.017	-0.037	0.031
Interference x Age	0.020	0.021	-0.021	0.061
Interference x ICAR	-0.008	0.022	-0.051	0.035
Interference x Gender 1	-0.024	0.075	-0.172	0.123
Interference x Gender 2	-0.015	0.075	-0.163	0.132
Reward condition x Transition x Traits	-0.009	0.015	-0.039	0.020
Reward condition x Transition x Order	-0.015	0.016	-0.047	0.017
**Reward condition x Transition x Task**	**-0.041**	**0.016**	**-0.073**	**-0.009**
Reward condition x Transition x Age	-0.019	0.016	-0.050	0.013
Reward condition x Transition x ICAR	0.025	0.017	-0.008	0.058
Reward condition x Transition x Gender 1	-0.046	0.059	-0.162	0.070
Reward condition x Transition x Gender 2	-0.057	0.059	-0.173	0.059
Reward condition x Interference x Traits	-0.008	0.015	-0.038	0.021
Reward condition x Interference x Order	-0.017	0.016	-0.049	0.015
Reward condition x Interference x Task	0.004	0.016	-0.028	0.036
Reward condition x Interference x Age	-0.006	0.016	-0.038	0.026
Reward condition x Interference x ICAR	-0.017	0.017	-0.051	0.016
Reward condition x Interference x Gender 1	-0.011	0.059	-0.127	0.103
Reward condition x Interference x Gender 2	-0.006	0.059	-0.122	0.109
Transition x Interference x Traits	0.007	0.015	-0.023	0.036
Transition x Interference x Order	-0.002	0.016	-0.033	0.030
Transition x Interference x Task	-0.002	0.016	-0.034	0.030
**Transition x Interference x Age**	**-0.036**	**0.016**	**-0.067**	**-0.005**
Transition x Interference x ICAR	0.012	0.017	-0.021	0.046
Transition x Interference x Gender 1	0.045	0.060	-0.074	0.163
Transition x Interference x Gender 2	-0.004	0.060	-0.124	0.114

Appendix H

Table H1

Group Analysis Free Choice Reaction Time

Coefficient	b	Est. Error	l-95% CI	u-95% CI
**Intercept**	**6.786**	**0.034**	**6.718**	**6.852**
Reward condition	-0.011	0.008	-0.026	0.004
**Transition**	**-0.020**	**0.005**	**-0.029**	**-0.011**
Interference	-0.005	0.003	-0.012	0.001
**Group**	**0.057**	**0.023**	**0.013**	**0.101**
Order	-0.029	0.016	-0.060	0.002
**Task**	**-0.020**	**0.004**	**-0.027**	**-0.013**
Age	0.017	0.016	-0.015	0.049
**ICAR**	**-0.049**	**0.016**	**-0.081**	**-0.017**
Gender 1	-0.066	0.037	-0.139	0.006
Gender 2	0.042	0.036	-0.029	0.113
Reward condition x Transition	0.001	0.003	-0.005	0.007
**Reward condition x Interference**	**-0.006**	**0.003**	**-0.012**	**-0.001**
Transition x Interference	0.002	0.003	-0.004	0.008
Reward condition x Group	-0.005	0.005	-0.015	0.006
**Reward condition x Order**	**0.043**	**0.004**	**0.036**	**0.051**
Reward condition x Task	0.002	0.001	0.000	0.005
Reward condition x Age	-0.002	0.004	-0.009	0.006
Reward condition x ICAR	-0.002	0.004	-0.010	0.005
Reward condition x Gender 1	0.002	0.009	-0.015	0.019
Reward condition x Gender 2	0.004	0.008	-0.012	0.020
Transition x Group	-0.002	0.003	-0.009	0.004
Transition x Order	0.004	0.002	-0.001	0.008
Transition x Task	0.001	0.001	-0.002	0.004
Transition x Age	-0.003	0.002	-0.007	0.002
**Transition x ICAR**	**0.007**	**0.002**	**0.003**	**0.012**
Transition x Gender 1	-0.008	0.005	-0.018	0.002
**Transition x Gender 2**	**-0.010**	**0.005**	**-0.020**	**-0.001**
Interference x Group	0.002	0.002	-0.003	0.006
Interference x Order	0.000	0.002	-0.003	0.003
Interference x Task	0.000	0.001	-0.003	0.003
Interference x Age	-0.001	0.002	-0.004	0.002
Interference x ICAR	-0.001	0.002	-0.004	0.002
Interference x Gender 1	0.000	0.004	-0.007	0.007
Interference x Gender 2	-0.001	0.004	-0.008	0.006
Reward condition x Transition x Group	0.000	0.002	-0.004	0.004
Reward condition x Transition x Order	-0.002	0.001	-0.005	0.000
Reward condition x Transition x Task	-0.001	0.001	-0.004	0.001
Reward condition x Transition x Age	0.002	0.001	0.000	0.005
Reward condition x Transition x ICAR	0.002	0.001	-0.001	0.004
Reward condition x Transition x Gender 1	0.000	0.003	-0.007	0.006
Reward condition x Transition x Gender 2	-0.003	0.003	-0.009	0.003
Reward condition x Interference x Group	-0.003	0.002	-0.007	0.001
Reward condition x Interference x Order	0.001	0.001	-0.001	0.004
Reward condition x Interference x Task	0.001	0.001	-0.002	0.004
Reward condition x Interference x Age	-0.003	0.001	-0.005	0.000
Reward condition x Interference x ICAR	0.000	0.001	-0.003	0.002
Reward condition x Interference x Gender 1	0.004	0.003	-0.002	0.010
Reward condition x Interference x Gender 2	0.005	0.003	-0.001	0.011
Transition x Interference x Group	0.001	0.002	-0.003	0.005
Transition x Interference x Order	0.000	0.001	-0.003	0.003
Transition x Interference x Task	-0.002	0.001	-0.005	0.001
Transition x Interference x Age	0.000	0.001	-0.003	0.003
Transition x Interference x ICAR	0.000	0.001	-0.003	0.002
Transition x Interference x Gender 1	0.000	0.003	-0.006	0.006
Transition x Interference x Gender 2	0.000	0.003	-0.006	0.006

Table H2

Trait Analysis Free Choice Reaction Time

Coefficient	b	Est. Error	l-95% CI	u-95% CI
**Intercept**	**6.721**	**0.056**	**6.611**	**6.832**
Reward condition	0.004	0.013	-0.020	0.029
**Transition**	**-0.016**	**0.007**	**-0.031**	**-0.002**
**Interference**	**-0.015**	**0.005**	**-0.025**	**-0.004**
Traits	-0.001	0.016	-0.032	0.029
Order	-0.017	0.017	-0.051	0.017
**Task**	**-0.022**	**0.004**	**-0.029**	**-0.014**
Age	0.024	0.017	-0.011	0.057
**ICAR**	**-0.041**	**0.018**	**-0.076**	**-0.007**
Gender 1	-0.044	0.058	-0.157	0.068
Gender 2	0.063	0.058	-0.050	0.177
Reward condition x Transition	-0.002	0.005	-0.012	0.007
Reward condition x Interference	-0.005	0.004	-0.013	0.004
Transition x Interference	0.005	0.005	-0.004	0.014
Reward condition x Traits	0.002	0.004	-0.005	0.008
**Reward condition x Order**	**0.041**	**0.004**	**0.033**	**0.048**
Reward condition x Task	0.002	0.001	0.000	0.005
Reward condition x Age	-0.002	0.004	-0.010	0.005
Reward condition x ICAR	-0.001	0.004	-0.009	0.007
Reward condition x Gender 1	-0.010	0.013	-0.036	0.015
Reward condition x Gender 2	-0.007	0.013	-0.033	0.019
Transition x Traits	0.001	0.002	-0.003	0.005
Transition x Order	0.004	0.002	-0.001	0.008
Transition x Task	0.002	0.001	-0.001	0.005
Transition x Age	-0.003	0.002	-0.007	0.002
**Transition x ICAR**	**0.008**	**0.002**	**0.003**	**0.013**
Transition x Gender 1	-0.009	0.008	-0.024	0.006
Transition x Gender 1	-0.013	0.008	-0.028	0.002
Interference x Traits	-0.001	0.002	-0.003	0.002
Interference x Order	0.001	0.002	-0.002	0.004
Interference x Task	0.001	0.001	-0.002	0.003
Interference x Age	-0.001	0.002	-0.005	0.002
Interference x ICAR	-0.002	0.002	-0.005	0.002
Interference x Gender 1	0.007	0.006	-0.004	0.018
Interference x Gender 2	0.006	0.006	-0.005	0.017
Reward condition x Transition x Traits	0.001	0.001	-0.002	0.003
Reward condition x Transition x Order	-0.003	0.002	-0.006	0.000
Reward condition x Transition x Task	-0.001	0.001	-0.003	0.002
Reward condition x Transition x Age	0.001	0.002	-0.002	0.004
Reward condition x Transition x ICAR	0.002	0.002	-0.001	0.005
Reward condition x Transition x Gender 1	0.003	0.005	-0.006	0.013
Reward condition x Transition x Gender 2	0.000	0.005	-0.009	0.010
Reward condition x Interference x Traits	0.000	0.001	-0.003	0.002
Reward condition x Interference x Order	0.001	0.001	-0.001	0.004
Reward condition x Interference x Task	0.001	0.001	-0.002	0.004
Reward condition x Interference x Age	-0.001	0.001	-0.004	0.002
Reward condition x Interference x ICAR	-0.001	0.001	-0.003	0.002
Reward condition x Interference x Gender 1	0.004	0.005	-0.005	0.013
Reward condition x Interference x Gender 2	0.007	0.005	-0.002	0.016
Transition x Interference x Traits	0.000	0.001	-0.003	0.002
Transition x Interference x Order	0.001	0.001	-0.001	0.004
Transition x Interference x Task	-0.002	0.001	-0.005	0.001
Transition x Interference x Age	0.000	0.001	-0.003	0.002
Transition x Interference x ICAR	0.000	0.001	-0.003	0.002
Transition x Interference x Gender 1	-0.005	0.005	-0.014	0.005
Transition x Interference x Gender 2	-0.005	0.005	-0.014	0.004

Table H3

Group Analysis Cued Choice Reaction Time

Coefficient	b	Est. Error	l-95% CI	u-95% CI
**Intercept**	**6.440**	**0.039**	**6.365**	**6.517**
Reward condition	-0.011	0.009	-0.028	0.006
**Transition**	**-0.054**	**0.005**	**-0.064**	**-0.044**
Interference	-0.004	0.004	-0.012	0.004
**Group**	**0.069**	**0.027**	**0.017**	**0.122**
**Order**	**-0.040**	**0.019**	**-0.076**	**-0.004**
**Task**	**-0.037**	**0.005**	**-0.047**	**-0.027**
Age	0.029	0.019	-0.008	0.066
**ICAR**	**-0.072**	**0.019**	**-0.109**	**-0.034**
**Gender 1**	**-0.102**	**0.042**	**-0.184**	**-0.019**
Gender 2	0.052	0.042	-0.030	0.133
Reward condition x Transition	0.001	0.004	-0.007	0.008
Reward condition x Interference	-0.007	0.004	-0.014	0.001
Transition x Interference	0.002	0.004	-0.005	0.010
Reward condition x Group	-0.006	0.006	-0.017	0.006
**Reward condition x Order**	**0.057**	**0.004**	**0.049**	**0.065**
Reward condition x Task	0.000	0.002	-0.004	0.003
Reward condition x Age	-0.002	0.004	-0.011	0.006
Reward condition x ICAR	-0.003	0.004	-0.011	0.005
Reward condition x Gender 1	-0.003	0.010	-0.022	0.016
Reward condition x Gender 2	0.009	0.009	-0.010	0.028
Transition x Group	-0.003	0.004	-0.010	0.004
Transition x Order	-0.002	0.003	-0.006	0.003
**Transition x Task**	**-0.011**	**0.002**	**-0.015**	**-0.007**
Transition x Age	-0.001	0.003	-0.006	0.004
Transition x ICAR	-0.001	0.003	-0.007	0.004
**Transition x Gender 1**	**0.013**	**0.006**	**0.001**	**0.024**
Transition x Gender 1	0.011	0.006	0.000	0.022
Interference x Group	0.002	0.003	-0.004	0.007
Interference x Order	0.000	0.002	-0.004	0.004
Interference x Task	0.000	0.002	-0.004	0.004
Interference x Age	0.001	0.002	-0.003	0.005
Interference x ICAR	0.001	0.002	-0.003	0.005
Interference x Gender 1	0.000	0.005	-0.009	0.009
Interference x Gender 2	0.006	0.004	-0.003	0.014
**Reward condition x Transition x Group**	**0.008**	**0.003**	**0.002**	**0.013**
**Reward condition x Transition x Order**	**-0.008**	**0.002**	**-0.011**	**-0.004**
Reward condition x Transition x Task	-0.002	0.002	-0.006	0.001
Reward condition x Transition x Age	0.002	0.002	-0.002	0.006
Reward condition x Transition x ICAR	0.003	0.002	-0.001	0.007
Reward condition x Transition x Gender 1	0.005	0.004	-0.004	0.013
Reward condition x Transition x Gender 2	0.005	0.004	-0.004	0.013
Reward condition x Interference x Group	0.002	0.003	-0.003	0.007
Reward condition x Interference x Order	0.003	0.002	-0.001	0.007
Reward condition x Interference x Task	-0.001	0.002	-0.004	0.003
Reward condition x Interference x Age	0.000	0.002	-0.004	0.004
Reward condition x Interference x ICAR	0.002	0.002	-0.002	0.005
**Reward condition x Interference x Gender 1**	**0.008**	**0.004**	**0.000**	**0.017**
Reward condition x Interference x Gender 2	0.006	0.004	-0.002	0.014
Transition x Interference x Group	0.000	0.003	-0.005	0.006
Transition x Interference x Order	0.002	0.002	-0.002	0.005
Transition x Interference x Task	0.001	0.002	-0.002	0.005
Transition x Interference x Age	0.003	0.002	-0.001	0.007
Transition x Interference x ICAR	-0.001	0.002	-0.004	0.003
Transition x Interference x Gender 1	-0.005	0.004	-0.014	0.003
Transition x Interference x Gender 2	0.005	0.004	-0.004	0.013

Table H4

Trait Analysis Cued Choice Reaction Time

Coefficient	b	Est. Error	l-95% CI	u-95% CI
**Intercept**	**6.407**	**0.058**	**6.295**	**6.520**
Reward condition	-0.003	0.013	-0.029	0.024
**Transition**	**-0.030**	**0.008**	**-0.046**	**-0.015**
Interference	0.000	0.006	-0.012	0.013
Traits	-0.003	0.016	-0.035	0.029
Order	-0.025	0.017	-0.059	0.010
**Task**	**-0.034**	**0.005**	**-0.043**	**-0.025**
**Age**	**0.036**	**0.018**	**0.002**	**0.071**
**ICAR**	**-0.061**	**0.018**	**-0.097**	**-0.026**
Gender 1	-0.008	0.059	-0.125	0.108
**Gender 2**	**0.127**	**0.060**	**0.008**	**0.243**
Reward condition x Transition	0.002	0.006	-0.009	0.013
Reward condition x Interference	-0.001	0.006	-0.013	0.010
Transition x Interference	0.004	0.006	-0.007	0.015
**Reward condition x Traits**	**-0.008**	**0.004**	**-0.015**	**0.000**
**Reward condition x Order**	**0.046**	**0.004**	**0.038**	**0.054**
Reward condition x Task	0.000	0.002	-0.003	0.004
Reward condition x Age	-0.003	0.004	-0.011	0.005
Reward condition x ICAR	-0.001	0.004	-0.009	0.007
Reward condition x Gender 1	-0.005	0.014	-0.033	0.022
Reward condition x Gender 2	0.006	0.014	-0.021	0.034
Transition x Traits	0.000	0.002	-0.005	0.004
Transition x Order	-0.001	0.002	-0.006	0.004
**Transition x Task**	**-0.008**	**0.002**	**-0.011**	**-0.004**
Transition x Age	-0.001	0.003	-0.006	0.004
Transition x ICAR	-0.001	0.003	-0.006	0.004
Transition x Gender 1	-0.003	0.008	-0.019	0.013
Transition x Gender 1	-0.004	0.008	-0.020	0.012
Interference x Traits	0.000	0.002	-0.003	0.004
Interference x Order	-0.001	0.002	-0.005	0.003
Interference x Task	0.000	0.002	-0.004	0.003
Interference x Age	0.000	0.002	-0.003	0.004
Interference x ICAR	0.001	0.002	-0.003	0.005
Interference x Gender 1	-0.007	0.007	-0.020	0.005
Interference x Gender 2	0.000	0.007	-0.013	0.013
Reward condition x Transition x Traits	0.002	0.002	-0.001	0.005
**Reward condition x Transition x Order**	**-0.005**	**0.002**	**-0.009**	**-0.002**
Reward condition x Transition x Task	-0.003	0.002	-0.006	0.001
Reward condition x Transition x Age	0.001	0.002	-0.003	0.004
Reward condition x Transition x ICAR	0.001	0.002	-0.002	0.005
Reward condition x Transition x Gender 1	-0.002	0.006	-0.014	0.009
Reward condition x Transition x Gender 2	-0.006	0.006	-0.018	0.005
Reward condition x Interference x Traits	-0.001	0.002	-0.004	0.002
Reward condition x Interference x Order	0.003	0.002	-0.001	0.006
Reward condition x Interference x Task	0.000	0.002	-0.003	0.003
Reward condition x Interference x Age	0.000	0.002	-0.004	0.003
Reward condition x Interference x ICAR	0.002	0.002	-0.002	0.005
Reward condition x Interference x Gender 1	0.002	0.006	-0.009	0.014
Reward condition x Interference x Gender 2	0.000	0.006	-0.011	0.012
Transition x Interference x Traits	-0.001	0.002	-0.004	0.002
Transition x Interference x Order	0.000	0.002	-0.004	0.003
Transition x Interference x Task	0.000	0.002	-0.003	0.003
Transition x Interference x Age	0.002	0.002	-0.002	0.005
Transition x Interference x ICAR	0.000	0.002	-0.004	0.004
Transition x Interference x Gender 1	-0.006	0.006	-0.018	0.005
Transition x Interference x Gender 2	0.002	0.006	-0.010	0.013
